# Integration of Gaussian process regression and K means clustering for enhanced short term rainfall runoff modeling

**DOI:** 10.1038/s41598-025-91339-8

**Published:** 2025-03-03

**Authors:** Ozgur Kisi, Salim Heddam, Kulwinder Singh Parmar, Andrea Petroselli, Christoph Külls, Mohammad Zounemat-Kermani

**Affiliations:** 1 Department of Civil Engineering, Technische Hochschule Lübeck, 23562 Lübeck, Germany; 2 Department of Civil Engineering, Ilia State University, 0162 Tbilisi, Georgia; 3 School of Civil, Environmental and Architectural Engineering, Korea University, Seoul, 02841 South Korea; 4 Faculty of Science, Agronomy Department, Hydraulics Division, University 20 Août 1955 Skikda, Route El Hadaik, BP 26, Skikda, Algeria; 5 Department of Mathematics, IKG Punjab Technical University, Jalandhar, Kapurthala India; 6 Department of Agriculture and Forest Sciences (DAFNE), University of Tuscia, Viterbo, Italy; 7 Civil Engineering Department, Shahid Bahonar University of Kerman, Kerman, Iran

**Keywords:** Rainfall-runoff modelling, Gaussian process regression, K-means clustering, Short-term streamflow prediction, Orgeval watershed, Principal component regression, Hydrology, Mathematics and computing

## Abstract

Accurate rainfall-runoff modeling is crucial for effective watershed management, hydraulic infrastructure safety, and flood mitigation. However, predicting rainfall-runoff remains challenging due to the nonlinear interplay between hydro-meteorological and topographical variables. This study introduces a hybrid Gaussian process regression (GPR) model integrated with K-means clustering (GPR-K-means) for short-term rainfall-runoff forecasting. The Orgeval watershed in France serves as the study area, providing hourly precipitation and streamflow data spanning 1970–2012. The performance of the GPR-K-means model is compared with standalone GPR and principal component regression (PCR) models across four forecasting horizons: 1-hour, 6-hour, 12-hour, and 24-hour ahead. The results reveal that the GPR-K-means model significantly improves forecasting accuracy across all lead times, with a Nash-Sutcliffe Efficiency (NSE) of approximately 0.999, 0.942, 0.891, and 0.859 for 1-hour, 6-hour, 12-hour, and 24-hour forecasts, respectively. These results outperform other ML models, such as Long Short-Term Memory, Support Vector Machines, and Random Forest, reported in the literature. The GPR-K-means model demonstrates enhanced reliability and robustness in hourly streamflow forecasting, emphasizing its potential for broader application in hydrological modeling. Furthermore, this study provides a novel methodology for combining clustering and Bayesian regression techniques in surface hydrology, contributing to more accurate and timely flood prediction.

## Introduction

The recent concerns of global warming and climate change, together with limits on water access in various locations, pose a challenge to hydrological and water resource sustainability. In this context, accurate rainfall-runoff modeling is critical for flood mitigation, agriculture, watershed management, soil erosion control, and hydraulic and irrigation structure safety^1^. Furthermore, precise runoff prediction is critical for conserving water supplies while minimizing rural and urban infrastructure damage. Predicting rainfall-runoff is a complex undertaking due to the influence of various hydro-meteorological variables. In addition to hydro-meteorological elements, the impact of watershed topography and characteristics (e.g., shape, vegetation, slope, altitude, soil type, soil water retention, percentage and land cover) on runoff production and concentration makes it a complex and nonlinear natural process. In this regard, throughout the last few decades, hydrologists have offered and developed various methodologies and approaches to dealing with the rainfall-runoff process, including physically-based^2^ and conceptual^3–5^ models.

In some watersheds, where climatic and hydrometric stations are scarce or poorly constructed, gathering the exact meteorological and topographical input data for these techniques can be challenging or impossible. However, most regions have access to essential meteorological and hydrological variables, including temperature, precipitation, and stream flow, because of synaptic stations and hydrometric monitoring. As a result, in recent decades, researchers have attempted to create empirical time series computer-aided models to correlate the influence of observed precipitation (and, in some cases, current discharge) with forecasted runoff. While data-driven models can offer powerful predictive capabilities, their effectiveness and cost-efficiency vary depending on the availability of quality data and computational resources. Nonetheless, due to the complexity and highly nonlinear relationship between input variables (such as precipitation) and output variables (here, runoff), novel data-driven models are currently being developed to better capture the complex behavior of the rainfall-runoff process.

Due to their structural flexibility and capacity to capture nonlinear behavior, soft computing approaches and Machine Learning (ML) models have grown in popularity in many rainfall-runoff modeling studies over the last few years^6,7^. Instances include the applications of different types of conventional and modern Artificial Neural Networks, ANNs (e.g., MLPNN, GRNN, ELM, & LSTM)^8–10^, Deep Learning (DL) models^11,12^, Fuzzy Inference Systems (FIS)^13^, k-nearest neighbors (KNN) algorithm^14^, Relevance Vector Machines (RVM)^15^, Support Vector Machines (SVMs)^16–18^, Genetic Programming (GP, GEP) models^19^, Tree-based algorithms (e.g., M5 model, MARS, CART, & CHAID)^20–22^, and ensemble modeling (e.g., RF, XGBoost)^23,24^.

While ML models have demonstrated strong predictive capabilities in rainfall-runoff modeling, they have limitations. Common challenges in interpretability, uncertainty quantification, and high data requirements often limit their applicability, particularly in data-sparse regions^6,25,26^. Additionally, their black-box nature complicates interpretability, which limits their ability to enhance our understanding of physical hydrological processes^1,13^. Uncertainty quantification also presents a challenge, as ML models like neural networks and support vector machines often lack robust approaches to quantify uncertainty, which is essential for reliable hydrological forecasting^25,27^. Furthermore, ML models are susceptible to overfitting, especially in hydrological studies with limited or imbalanced data, which reduces generalizability across different catchments^28,29^.

Physically based models such as Soil & Water Assessment Tool (SWAT) and HEC-HMS have been widely used for rainfall-runoff modeling. However, their reliance on extensive input parameters and calibration requirements often limits their applicability in data-scarce regions. To overcome these limitations, data-driven machine learning approaches have been increasingly adopted^30,31^. However, conceptual and physically based models retain advantages regarding interpretability and generalizability, especially in data-sparse regions. For example, Vilaseca et al.^23^ investigated the capabilities of ML techniques in rainfall-runoff modeling using the Random Forest (RF) model and a conceptual model SWAT. The RF model was more accurate for the calibration and training sets, whereas the SWAT model performed better for both the validation and testing sets. Furthermore, the RF surpassed SWAT regarding the computational time required for adequate calibration and training. In another study, Sayed et al.^13^ investigated and compared the abilities of HEC-HMS (as a physical-based numerical model) and TOPMODEL (as a conceptual model) to the GEP (Gene Expression Programming) and ANFIS (adaptive neural fuzzy inference system) to predict the rainfall-runoff process based on long term historical datasets in northern Iraq. The outcomes of the study revealed that the applied ML models outperform traditional forecasting techniques. Other studies have also stated the superiority of ML models over physically-based models^19,22^.

The Gaussian process regression (GPR) approach is a newly used kernel-based machine learning algorithm in hydrology and water sciences. This methodology is based on a kernel method that parameterizes the unknown function in the feature space by adjusting the weighted sum of nonlinear basis functions, just like the SVMs and RVMs. In contrast to SVMs and RVMs, the GPR analyzes data to estimate the posterior distribution of weights using a Bayesian framework, assuming that the weights are random variables. The GPR has only been used in hydrometeorology^32,33^, precipitation^34,35^, and surface hydrology^28^ modeling thus far. Sun et al.^28^ employed the GPR to perform probabilistic streamflow forecasts on six hydrometric stations in the United States. They argued that the GPR technique provides a simple and adaptable hierarchical Bayesian framework for predicting the distribution of streamflow. The GPR outperforms both linear regression and ANN models in most circumstances. In another study, Sun et al.^29^ used integrated ML simulative techniques, including ANN, RF, and GPR models, to estimate streamflow with multi-scale factors in southwest China. The implementation of the RF-GPR model produced the best results. Ehteram et al.^25^ used an integrative deep learning model that incorporated the convolutional neural network (CONN), SVM, and GPR, known as the CONN-SVM-GPR, to forecast daily and monthly rainfall data in Malaysia’s Terengganu River Basin. The suggested CONN-SVM-GPR model outperformed the separate CONN, SVM, and GPR models. Several studies have integrated clustering techniques into ML-based models to enhance predictive performance^36,37^. For instance, Wu et al.^27^ applied a self-organizing map (SOM) to preprocess hydrological data before using a support vector regression model, achieving notable improvements in extreme streamflow predictions. However, unlike SOM, which relies on unsupervised learning and neural network structures, K-means clustering offers a computationally efficient alternative that partitions data into distinct, non-overlapping clusters, making it well-suited for Gaussian Process Regression (GPR). This study extends these earlier efforts by integrating K-means with GPR, a Bayesian regression technique that provides probabilistic predictions, thus improving both accuracy and uncertainty quantification in short-term rainfall-runoff forecasting.

According to the findings and conclusions of the associated literature analysis, ML models are a promising alternative for simulating rainfall-runoff systems utilizing time series data. Moreover, the integrative application of GPR combined with a clustering technique might enhance the accuracy and robustness of the developed GPR model^25,28^.

Despite progress in using ML models for rainfall-runoff predictions, many challenges remain—especially when accurately capturing the complex and nonlinear nature of short-term streamflow. This is particularly difficult in areas where data is limited. While these ML models often perform well, they don’t always make the best use of patterns in the data that could improve accuracy. Most existing studies don’t explore how combining clustering techniques, like K-means, with regression models might enhance predictions. Our study aims to fill this gap by introducing a new hybrid approach that combines GPR with K-means clustering, namely GPR-K-means^25,29^. This approach offers a better way to capture the complexity of streamflow patterns over short time frames. By comparing the hybrid model to simpler GPR and Principal Component Regression (PCR) models, we highlight how adding clustering can improve accuracy in predicting streamflow, addressing a need that hasn’t been fully explored in earlier research.

This study introduces a novel hybrid approach that integrates Gaussian Process Regression (GPR) with K-means clustering to improve short-term rainfall-runoff forecasting. Unlike standalone ML models, which often struggle with uncertainty quantification and data efficiency, the proposed GPR-K-means model enhances predictive accuracy by leveraging the strengths of both Bayesian regression and clustering techniques. While previous studies have employed clustering methods separately or combined GPR with other ML techniques, our approach uniquely incorporates K-means within the GPR framework, allowing structured data segmentation that enhances model performance. This method is particularly advantageous for short-term forecasting across multiple lead times, demonstrating superior accuracy and robustness compared to existing approaches. By addressing the challenges associated with interpretability and uncertainty in hydrological predictions, this study contributes a significant advancement in data-driven rainfall-runoff modeling.

## Materials and methods

### Case study: the Orgeval watershed

**Fig. 1:**
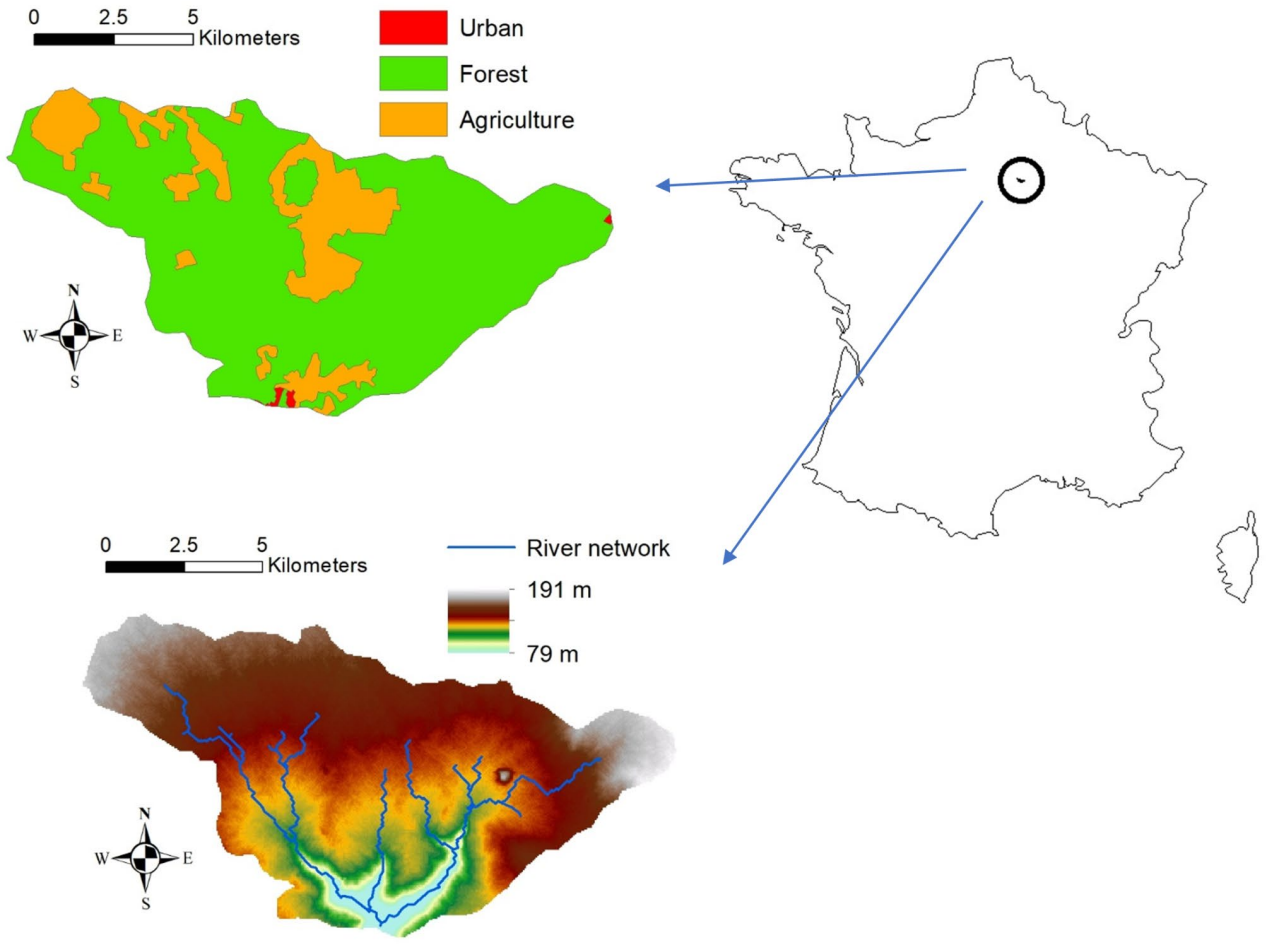
The study area—Orgeval watershed.

The present study examines the Orgeval watershed (Fig. 1) in France. This watershed is a pertinent case study for understanding hydrological processes within a specific geographical context. Positioned within the Seine basin, the Orgeval Watershed lies approximately 70 km east of Paris, playing an important role in regional hydrology and water resource management. With an annual precipitation rate averaging around 700 mm, the climate of the Orgeval Watershed is categorized as semi-oceanic, characterized by moderate rainfall patterns influenced by its proximity to maritime sources.

Geologically, the soil composition of the Orgeval Watershed is characterized by the top layer primarily consisting of silt, while the sublayer is predominantly clay. This composition influences water retention, infiltration rates, and overall hydrological dynamics within the watershed. The land use within the basin is primarily agricultural, encompassing approximately 82% of the total surface area. The remaining land cover is distributed among woodland areas, constituting 17%, with urban zones and roads occupying a minor 1% of the total surface area.

A comprehensive dataset from 1970 to 2012 provides hourly observations of precipitation and discharge within the Orgeval Watershed, facilitating detailed analysis of hydrological trends and patterns over a significant period. This dataset, and a Digital Elevation Model at 50 m resolution, were sourced from the GIS ORACLE – CEMAGREF. Additionally, land cover data utilized in the study were retrieved from the CORINE (Coordination of Information on Environment) Database (2000)^38^.

The Orgeval watershed exhibits distinctive geomorphological characteristics that shape its hydrological behavior. Covering a contributing area of 106.1 km², the watershed’s boundaries are clearly delineated in this study for accurate hydrological modeling. At 2,425,657 N, 656,976 E in the NTF Lambert projection system, the outlet coordinates serve as critical reference points for spatial analysis and modeling efforts. The elevational profile of the watershed varies significantly, with minimum, average, and maximum elevations measured at 79 m, 156 m, and 191 m above average sea level, respectively.

Furthermore, the average slope of the basin is recorded at 1.9%, influencing surface runoff patterns and drainage dynamics. The main channel within the watershed spans a length of 13.1 km, serving as a primary conduit for water flow and discharge. Hydrological modeling efforts benefit from previously determined parameters such as the average Curve Number (CN) value (estimated at 76.5 from the original SCS look-up tables that link land use/soil type and CN values), which provides insights into the watershed’s hydrological response to precipitation events. Additionally, the concentration time (estimated at 10.3 h using Giandotti’s formula) offers valuable information regarding the temporal distribution of runoff within the watershed, as it helps to predict how quickly and efficiently precipitation will be transformed into surface runoff. Understanding the time of concentration allows for better flood risk assessment, watershed management, and the design of stormwater systems, as it highlights the relationship between rainfall intensity, duration, and the resulting flow patterns. For more details on the Orgeval River basin, reference can be made to Vilain et al.^39^.

### Principal component regression (PCR)

The two-step methodology is chosen to construct the PCR (principal component regression) model. The first step implements PCA (principal component analysis) model properties; in the second step, the regression model executes new explanatory variables with principal components. The PCR model has many new couplings; in our case, only the PCA model is applied to the explanatory variables^40,41^. The historical involvement of the present model facilitates a linear regression model between input and output variables^42,43^.

Let $X \in R^{n \times m}$ be the input data set; the total count of samples and variables are represented with *n* and *m*, respectively. Likewise, $Y \in R^{n \times l}$ means the output data set holds *n* observations, and the quality variable is denoted with *l*.

To develop the eigenvalue and loading matrices $\Lambda = {\text{diag}}\left( {\lambda_{1} ,\lambda_{2} ,\lambda_{3} ,.....\lambda_{m} } \right)$, SVD (singular value decomposition) was used for the covariance matrix $\frac{{X^{T} X}}{n - 1}$ ^35^. Here $P = \left[ {p_{1} ,p_{2} ,p_{3} , \ldots ,p_{m} } \right]$ is the loading matrix, covariance matrix is herewith simplified with the loading matrix. Power T means the transpose of the matrices.

1 $$\frac{{X^{T} X}}{n - 1} = \left[ {\begin{array}{*{20}l} {\overset{\lower0.5em\hbox{$\smash{\scriptscriptstyle\frown}$}}{P} } \hfill & {\tilde{P}} \hfill \\ \end{array} } \right]\left[ {\begin{array}{*{20}c} {\Lambda_{pc} } & 0 \\ 0 & {\Lambda_{res} } \\ \end{array} } \right]\left[ \begin{gathered} \overset{\lower0.5em\hbox{$\smash{\scriptscriptstyle\frown}$}}{P}^{T} \hfill \\ \tilde{P}^{T} \hfill \\ \end{gathered} \right] \approx \overset{\lower0.5em\hbox{$\smash{\scriptscriptstyle\frown}$}}{P} \Lambda_{pc} \overset{\lower0.5em\hbox{$\smash{\scriptscriptstyle\frown}$}}{P}^{T}$$

The process variables here are divided into residual subspace and principal subspace.

2 $$X = \overset{\lower0.5em\hbox{$\smash{\scriptscriptstyle\frown}$}}{X} + \tilde{X} = \overset{\lower0.5em\hbox{$\smash{\scriptscriptstyle\frown}$}}{T} \overset{\lower0.5em\hbox{$\smash{\scriptscriptstyle\frown}$}}{P}^{T} + \tilde{X}$$

In this equation, $Y^{T} \approx \phi X^{T}$ multiply both sides by $\frac{X}{n - 1}$, then $\frac{{Y^{T} X}}{n - 1} \approx \phi \frac{{X^{T} X}}{n - 1}$.

The regression coefficient is conveyed and simplifies the equations as:

3 $$\phi = \frac{{Y^{T} X\overset{\lower0.5em\hbox{$\smash{\scriptscriptstyle\frown}$}}{P} \Lambda_{pc}^{ - 1} \overset{\lower0.5em\hbox{$\smash{\scriptscriptstyle\frown}$}}{P}^{T} }}{n - 1} = \frac{{Y^{T} \left( {\Lambda_{pc}^{{\frac{ - 1}{2}}} \overset{\lower0.5em\hbox{$\smash{\scriptscriptstyle\frown}$}}{P}^{T} X^{T} } \right)^{T} \Lambda_{pc}^{{\frac{ - 1}{2}}} \overset{\lower0.5em\hbox{$\smash{\scriptscriptstyle\frown}$}}{P}^{T} }}{n - 1}$$

4 $$\overline{\phi } = \Lambda_{pc}^{{\frac{ - 1}{2}}} \overset{\lower0.5em\hbox{$\smash{\scriptscriptstyle\frown}$}}{P}^{T}$$

The PCR model can be attained by:

5 $$\overset{\lower0.5em\hbox{$\smash{\scriptscriptstyle\frown}$}}{y} = \phi x = \frac{{Y^{T} \left( {\Lambda_{pc}^{{\frac{ - 1}{2}}} \overset{\lower0.5em\hbox{$\smash{\scriptscriptstyle\frown}$}}{P}^{T} X^{T} } \right)^{T} \Lambda_{pc}^{{\frac{ - 1}{2}}} \overset{\lower0.5em\hbox{$\smash{\scriptscriptstyle\frown}$}}{P}^{T} }}{n - 1}x = \overline{\phi }\overline{x}$$

The SVD is reused, but here the $$\overline{\phi }$$ is used to simplify the Eq. (5). Here, $$\overline{x} = \Lambda_{pc}^{{\frac{ - 1}{2}}} \overset{\lower0.5em\hbox{$\smash{\scriptscriptstyle\frown}$}}{P}^{T} x$$ is the normalized input variable and $$\overline{\phi }$$ is the corresponding regression coefficient vector. $$\overset{\lower0.5em\hbox{$\smash{\scriptscriptstyle\frown}$}}{y}$$ is the output of the principal component regression model. From the Eq. (5) the decomposed outputs are given below.

6 $$\bar \phi = \frac{{{Y^T}{{\left( {\Lambda _{pc}^{\frac{{ - 1}}{2}}{{\overset{\lower0.5em\hbox{$\smash{\scriptscriptstyle\frown}$}}{P} }^T}{X^T}} \right)}^T}}}{{n - 1}} = \overset{\lower0.5em\hbox{$\smash{\scriptscriptstyle\frown}$}} {U} \left[ {\begin{matrix}{{D^{1/2}}} & 0 \\ \end{matrix} } \right]\left[ \begin{matrix} {{\overset{\lower0.5em\hbox{$\smash{\scriptscriptstyle\frown}$}} {V} }^T} \hfill \cr {{\tilde V}^T} \hfill \cr \end{matrix} \right] = \overset{\lower0.5em\hbox{$\smash{\scriptscriptstyle\frown}$}} {U} {D^{1/2}}{\overset{\lower0.5em\hbox{$\smash{\scriptscriptstyle\frown}$}}{V} ^T}$$

The mapping *y* into *x* is provided as given below.

7 $$\overline{y} = \overset{\lower0.5em\hbox{$\smash{\scriptscriptstyle\frown}$}}{V}^{T} \overline{x} = \overset{\lower0.5em\hbox{$\smash{\scriptscriptstyle\frown}$}}{V}^{T} \Lambda_{pc}^{{\frac{ - 1}{2}}} \overset{\lower0.5em\hbox{$\smash{\scriptscriptstyle\frown}$}}{P}^{T} x \sim N\left( {0,I_{l \times l} } \right)$$

Here, the critical variable vector for quality is $\overline{y}$. After this, *y* is predicted as follows.

8 $$\overset{\lower0.5em\hbox{$\smash{\scriptscriptstyle\frown}$}}{y} = \overline{\phi }\overline{x} = \overset{\lower0.5em\hbox{$\smash{\scriptscriptstyle\frown}$}}{U} D^{{{1 \mathord{\left/ {\vphantom {1 2}} \right. \kern-0pt} 2}}} \overset{\lower0.5em\hbox{$\smash{\scriptscriptstyle\frown}$}}{V}^{T} \Lambda_{pc}^{{\frac{ - 1}{2}}} \overset{\lower0.5em\hbox{$\smash{\scriptscriptstyle\frown}$}}{P}^{T} x = \overset{\lower0.5em\hbox{$\smash{\scriptscriptstyle\frown}$}}{U} D^{{{1 \mathord{\left/ {\vphantom {1 2}} \right. \kern-0pt} 2}}} \overline{y}$$

The prediction contributions of this PCR model are used to predict the short-term rainfall runoff in Orgeval watershed.

## Gaussian process regression (GPR)

The Gaussian process regression (GPR) model is a non-parametric supervised learning method mainly used to solve probabilistic classification and regression problems^44^. It is used to intricately identify the presence of hesitation in the model, which is further used to improve insight into the debris degradation process^43,45^. This GPR model is among the essential supervised learning models, particularly following Bayesian machine learning techniques. Its remarkable capacity to excel with small datasets without encountering underfitting or overfitting issues sets it apart. Its proficiency extends to examining datasets with nonlinear relationships among variables, leveraging probabilistic methodologies and posterior approximations. This flexibility is achieved by using a Gaussian prior distribution combined with a covariance function that adapts to patterns in the dataset, allowing for practical and robust analysis^46–48^. The versatility of the covariance function plays a pivotal role in Gaussian processes, enabling handling tasks characterized by diverse structures. Additionally, the standard properties of the GPR model make it a robust and versatile tool for statistical modeling. The covariance establishes the Gaussian process alongside the mean function $m(x)$.

9 $$m(x) = E\left( {f\left( x \right)} \right)$$

10 $$k\left( {x,x^{\prime}} \right) = E\left( {\left( {f\left( x \right) - m(x)} \right)\left( {f\left( {x^{\prime}} \right) - m(x^{\prime})} \right)} \right)$$

in the above equation, $k\left( {x,x^{\prime}} \right)$ represents the function of the covariance or kernel, which is assessed in the points $x$ and $x^{\prime}$. The Gaussian process function is defined below

11 $$f\left( x \right) \sim GP\left( {m(x),k\left( {x,x^{\prime}} \right)} \right)$$

The mean function holds a zero value, and the subsequent form can discern the connection between the input vector and the output target.

12 $$y = f\left( x \right) + \delta$$

The Gaussian white noise is represented with $\delta$ which is uncorrelated with mean value zero, function $f\left( x \right)$ and variance $\sigma^{2}$. The function $f\left( x \right)$ and dependent variable *y* having the Gaussian distribution and the set of the joint distribution of its finite observation is a Gaussian process:

13 $$y \sim GP\left( {m(x),k\left( {x,x^{\prime}} \right) + \sigma^{2} \gamma_{i\,j} } \right)$$

The Kronecker delta function is represented by $\gamma_{i\,j}$. Also, it is supposed that $y = [y_{1} ,y_{2} ,y_{3} ,....,y_{n} ]^{T}$ and $f = [f\left( {x_{1} } \right),f\left( {x_{2} } \right),f\left( {x_{3} } \right),....,f\left( {x_{n} } \right)]$ is a performance responsible upon the Gaussian process function that is

14 $$p\left( f \right) = N\left( {0,K} \right)$$

Here, *k* is the covariance matrix with the elements $k_{i,\,j} = k\left( {x_{i} ,x_{j} } \right)$

15 $$k = \left[ \begin{gathered} k\left( {x_{1} ,x_{1} } \right)\,\,k\left( {x_{1} ,x_{2} } \right).....k\left( {x_{1} ,x_{n} } \right) \hfill \\ k\left( {x_{2} ,x_{1} } \right)\,\,k\left( {x_{2} ,x_{2} } \right).....k\left( {x_{2} ,x_{n} } \right) \hfill \\ ................................................ \hfill \\ ................................................. \hfill \\ k\left( {x_{n} ,x_{1} } \right)\,\,k\left( {x_{n} ,x_{2} } \right).....k\left( {x_{n} ,x_{n} } \right) \hfill \\ \end{gathered} \right]$$

$k_{i}$ is the covariance between the values of the eigenfunctions $f\left( {x_{i} } \right)$ and $f\left( {x_{j} } \right)$. The GPR model is used to analyze the estimated distribution for the function values of the $f^{*}$ in the test point $X^{*} = [x^{*}_{1} ,x^{*}_{2} ,.....,x^{*}_{m} ]$. As per the description of the Gaussian process, the marginal distribution $p\left( f \right)$ is given by a Gaussian, as shown below.

16 $$p\left( f \right) = N\left( {\left. f \right|0,K} \right)$$

17 $$K_{y} = K + \sigma^{2} I$$

18 $$\left[ \begin{gathered} y \hfill \\ y^{*} \hfill \\ \end{gathered} \right] = \left( {\left[ \begin{gathered} f \hfill \\ f^{*} \hfill \\ \end{gathered} \right] + \left[ \begin{gathered} \delta \hfill \\ \delta^{*} \hfill \\ \end{gathered} \right]} \right)N\left( {0,\left[ \begin{gathered} K_{y} \,\,\,\,\,\,\,\,\,\,\,\,\,\,K^{*} \hfill \\ K_{n}^{T} \,\,\,\,\,\,K^{**} + \sigma^{2} \hfill \\ \end{gathered} \right]} \right)$$

here, $f^{*} = f^{*} \left( x \right)$ Eigen function for the variable $x^{*}$ and $\delta^{*}$ also

19 $$K^{*} = \left[ {k\left( {x^{*} ,x_{1} } \right)\,\,k\left( {x^{*} ,x_{2} } \right).....k\left( {x^{*} ,x_{n} } \right)} \right]^{T}$$

20 $$K^{**} = k\left( {x^{*} ,x^{*} } \right)$$

**Fig. 2:**
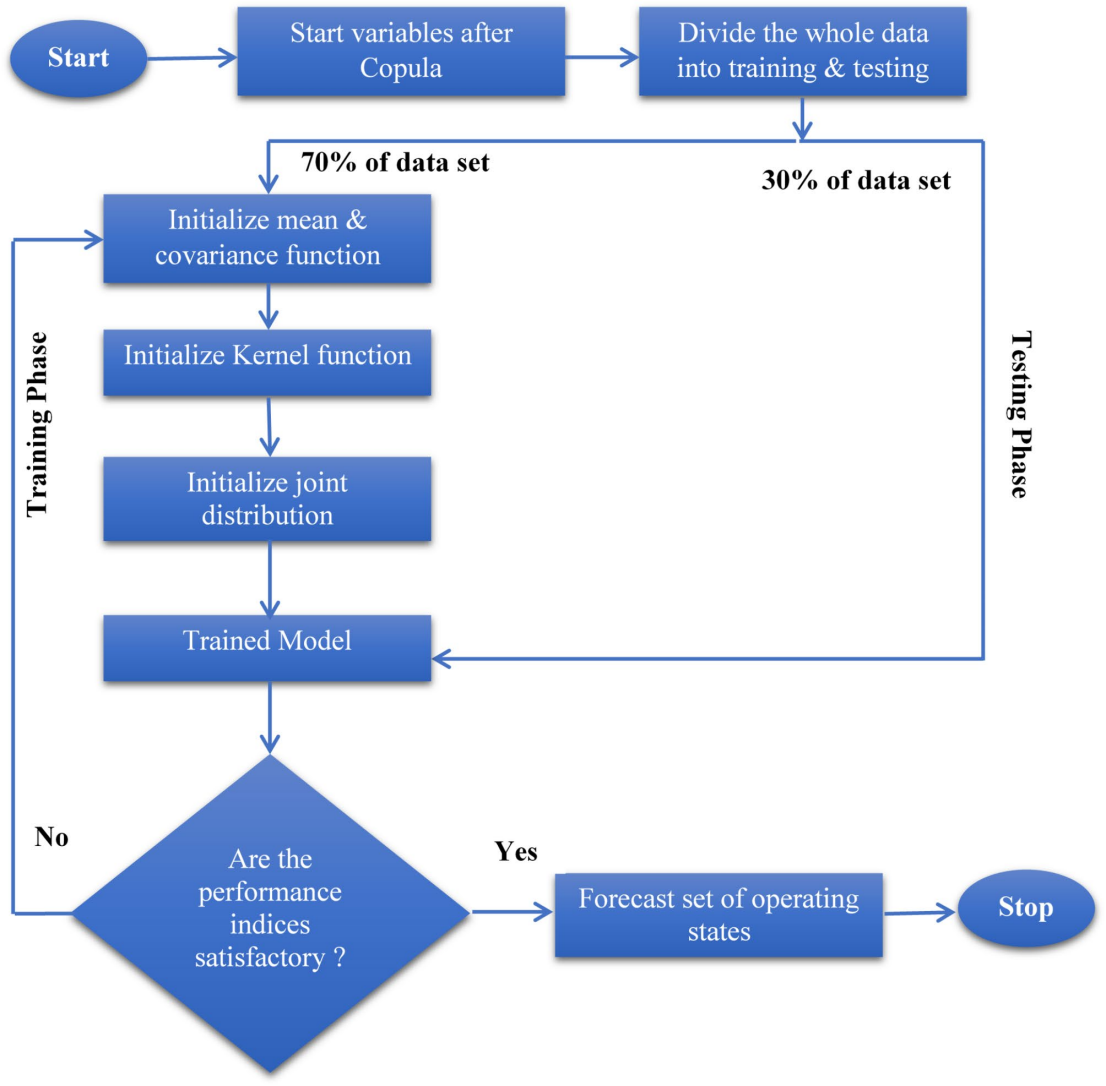
The flow chart of the GPR model.

Using the condition laws of the Gaussian^46^, the estimated distribution $p\left( {\left. {y^{*} } \right|y} \right)$ is a Gaussian distribution with the mean and variance $m\left( {{x^*}} \right) = {k^{*T}}k_y^{ - 1}y$. The covariance performance plays a vital role in the performance of the Gaussian process regression model. The flow chart of the GPR model is shown in Fig. 2.

### Gaussian process regression integrated with K-means clustering

In 1957, Stuart Lloyd first developed the K-means formulations; in 1967, Queen^49^ established the K-means applications. Again, in 1982, Lloyd^50^ introduced the vital application of K-means as an approximation to it. The partition-based clustering technique is the foundation for this K-means formulation and finds application across various research domains. As mentioned earlier, the distance matrix is pivotal in developing the K-means model. Specifically, the squared Euclidean distance between the row vector and the centroid of each cluster is utilized as a distance criterion. Initially, *k* initial seeds are considered, followed by comparing the Euclidean distance with each initial seed to determine the nearest adjoining cluster seed. This iteration is repeated until reaching an acceptable level or the most minor error value. The model’s accuracy performance is contingent upon the choice of initial seed and the number of clusters. This model organizes the objects depending upon features into K (+ ve) number of groups. The silhouette plot method is employed to analyze and validate data clusters. The dendrogram technique is applied to find the optimal cluster.

In the K-means system, an objective function *J,* and *J*_i_ is the Euclidean distance, *x*_k_ is the vector data, the number of clusters is *c*, *c*_*i*_ is the cluster center. An objective function is defined below.

21 $$J = \sum\limits_{i = 1}^{c} {J_{i} } = \sum\limits_{i = 1}^{c} {\left( {\sum\limits_{{k,x_{k} \in G_{i} }} {\left\| {x_{k} - c_{i} } \right\|}^{2} } \right)}$$

22 $$U = {\left[ {\begin{matrix}{{u_{11}}} \hfill & {{u_{12}}} \hfill & \cdots \hfill & {{u_{1n}}} \hfill \\ {{u_{21}}} \hfill & {{u_{22}}} \hfill & \cdots \hfill & {{u_{2n}}} \hfill \\ \cdot \hfill & \cdot \hfill & \cdots \hfill & \cdot \hfill \\ {{u_{c1}}} \hfill & {{u_{c2}}} \hfill & \cdots \hfill & {{u_{cn}}} \hfill \\ \end{matrix} } \right]_{c \times n}}$$

The cluster centers are assessed by $\left| {G_{i} } \right| = \sum\nolimits_{j = 1}^{n} {u_{ij} }$, the degrees of membership (1 or 0) are known based on the above matrix *U*. Minimization of objective function *J*. The output of K-means clustering shall have values 1 or 0, which suggests that data is from not cluster or cluster respectively.

23 $${u_{ij}} = \left\{ {\begin{array}{ll}1 & {{\text{if}}\,{{\left\| {{x_j} - {c_i}} \right\|}^2} \leqslant \,{{\left\| {{x_k} - {c_i}} \right\|}^2};\,\,{\text{for}}\,k \ne i} \\ 0 & {{\text{if}}\,{{\left\| {{x_j} - {c_i}} \right\|}^2} > \,{{\left\| {{x_k} - {c_i}} \right\|}^2}} \\ \end{array} } \right\}$$

where $$c_{i} = \frac{1}{{\left| {G_{i} } \right|}}\sum\nolimits_{{k,x_{k} \in G_{i} }} {x_{k} }$$.

**Fig. 3:**
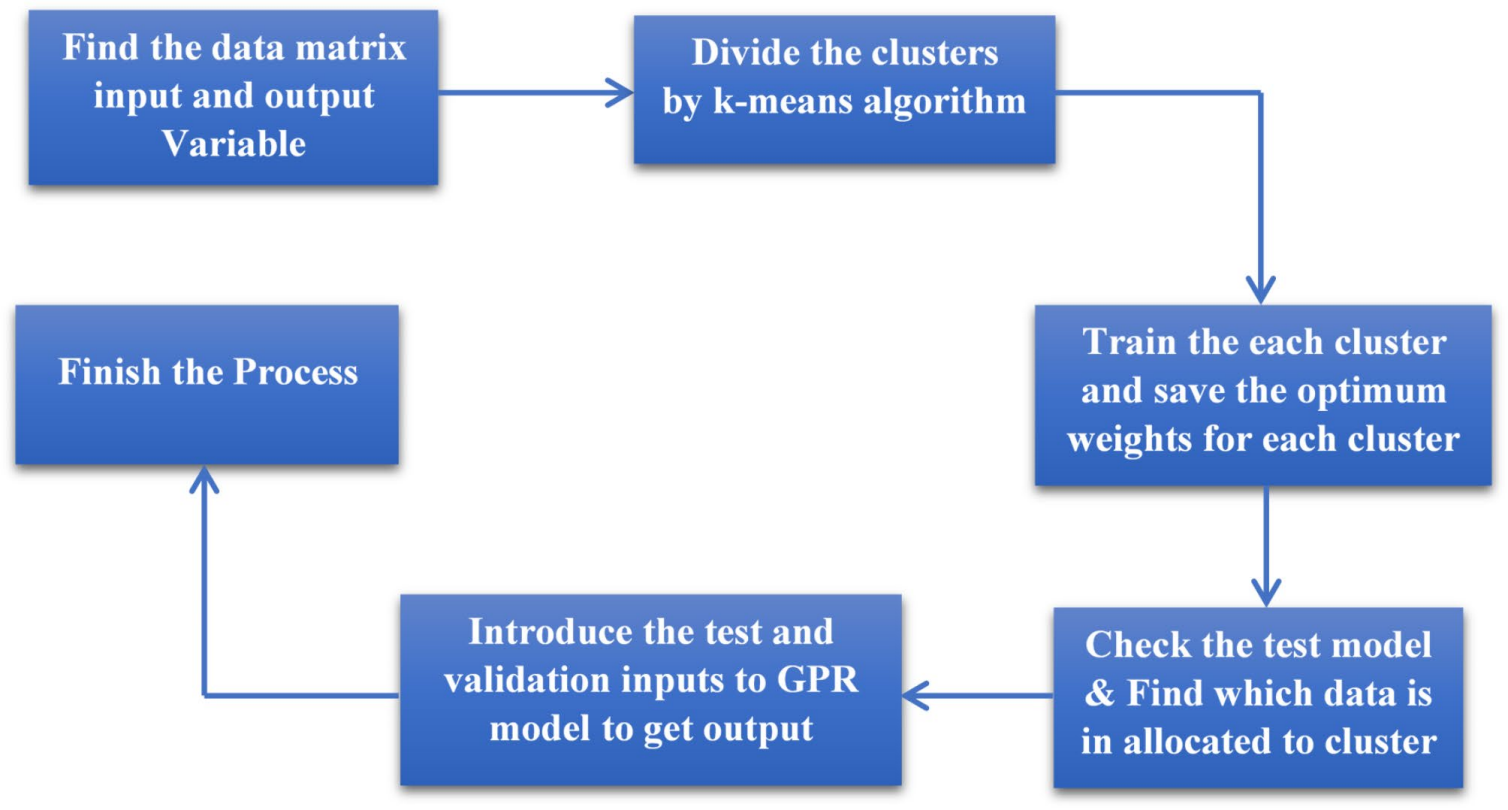
The flow chart of the proposed GPR-Kmeans model.

In the current research, the GPR model is integrated with the K-means algorithm for better prediction output with minimum error. An algorithm is established for integrating K-means clustering with the GPR model, and after coupling, the silhouette plot is used for validation and interpretation. This will help to graphically find each object in its own cluster. In the early stages, the regression method is applied to evaluate the most influential variables for the GPR model. Then, these clusters are trained independently, as discussed in the GPR model algorithm. This proposed model (GPR integrated with K-mean) performed better than the simple GPR and PCR models. The proposed coupled model developed the relationship between dependent and independent variables at each phase. This proposed integrated model is applied to forecast short-term rainfall runoff in the selected case study (see Fig. 3).

### Accuracy evaluation metrics

In this study, multiple evaluation metrics were employed to assess the performance of the rainfall-runoff models across various forecasting horizons. These metrics provide a holistic understanding of model accuracy, allowing for robust comparisons between models. Here, we describe each metric and its significance in evaluating predictive accuracy.

#### Taylor plot

A Taylor plot is a graphical representation used to summarize the performance of multiple models using three statistics: correlation coefficient (R), standard deviation, and root mean square error (RMSE). Developed by Taylor^51^, this plot compares multiple models against observed data. Points closer to the observed point on a Taylor plot indicate better model performance. The plot allows for easy visualization of how each model compares regarding variance and correlation, making it a valuable tool for model assessment.

#### Spider plot (radar plot)

A spider plot, also known as a radar plot, displays multiple metrics simultaneously for different models in a single, multi-axis chart^52^. Each axis represents a different evaluation metric, such as R, RMSE, Mean Absolute Error (MAE), and Nash-Sutcliffe Efficiency (NSE). This plot visually compares models across several metrics simultaneously, with larger “spikes” representing better performance. Spider plots are particularly useful for multi-objective assessments, where different metrics may weigh differently in the evaluation.

#### Correlation coefficient (R)

The correlation coefficient (R) measures the strength and direction of the linear relationship between observed and predicted values. Values close to 1 indicate a strong positive correlation, suggesting that the model captures the general trend of the observed data. A higher R value suggests that the model predictions are closely aligned with the observed values, demonstrating good model performance in capturing temporal dynamics^53^.

#### Root mean square error (RMSE)

RMSE is a commonly used metric for evaluating the accuracy of model predictions, calculated as the square root of the average of the squared differences between observed and predicted values. It measures the average error magnitude in the same units as the data. Lower RMSE values indicate better model accuracy, reflecting a smaller average deviation from observed values. RMSE is sensitive to significant errors, making it particularly useful when outliers are a concern^54^.

In addition to the above metrics, this study incorporates other statistical indicators such as Nash-Sutcliffe Efficiency (NSE) and Mean Absolute Error (MAE) to provide a more comprehensive assessment of model performance.

#### NSE

Measures how well the predicted data matches the observed data, with values closer to 1 indicating excellent model performance. This metric is handy in hydrological modeling.

#### MAE

Represents the average of the absolute differences between observed and predicted values, offering a straightforward interpretation of the average error magnitude without emphasizing significant errors as much as RMSE.

Combining these metrics provides a more nuanced view of model performance across various aspects, such as error magnitude, variance, and correlation. Visual tools like Taylor and spider plots enable quick, intuitive comparisons, making identifying the most suitable models for specific forecasting horizons easier.

## Results and discussion

Only a few studies have attempted to compare results between various ML models for multi-step ahead hourly streamflow forecasting. Here, we have compared the results of two available ML over the investigated watershed. We compared: (*i*) the Gaussian process regression (GPR), (*ii*) the principal component regression (PCR), and (*iii*) the GPR integrated with K-means clustering (GPR-K-means) with two and three clusters of rainfall (*2-P*,* 3-P*) and runoff (*2-Q*,* 3-Q*), respectively. First, among four input combinations, as depicted in Table 1, the best-input combination of rainfall (*P*) and runoff (*Q*) was examined and highlighted, and this first analysis of the present was conducted only one hour ahead of forecasting. The hourly trend of the rainfall and runoff datasets over the entire period of record was taken into account. Across all four input combinations, we have chosen to compare the models with only rainfall data measured at various previous lags, and the models that combine rainfall and runoff. This is particularly significant as the level of agreement between rainfalls at various lag-times is high.

**Table 1:** Training and validation statistics of ML methods in estimating 1-hour ahead streamflow.

Input combination	Validation				Testing			
	RMSE, m^3^/s	MAE, m^3^/s	NSE	*R* ^2^	RMSE, m^3^/s	MAE, m^3^/s	NSE	*R* ^2^
GPR								
* Pt-1*,* Pt-2*,* …*,* Pt-5*	1.433	0.649	0.112	0.136	0.751	0.464	− 0.100	0.058
* Pt-1*,* Pt-2*,* …*,* Pt-10*	1.366	0.628	0.192	0.225	0.735	0.446	− 0.055	0.113
* Pt-1*,* Pt-2*,* …*,* Pt-10*,* Qt*	0.166	0.027	0.988	0.988	0.088	0.014	0.985	0.985
* Pt-1*,* Pt-2*,* …*,* Pt-10*,* Qt*,* Qt-1*,*…*,* Qt-5*	**0.055**	**0.010**	**0.998**	**0.998**	**0.029**	**0.005**	**0.998**	**0.998**
PCR								
* Pt-1*,* Pt-2*,* …*,* Pt-5*	1.455	0.658	0.084	0.084	0.759	0.474	− 0.124	0.037
* Pt-1*,* Pt-2*,* …*,* Pt-10*	1.400	0.640	0.152	0.186	0.751	0.460	− 0.100	0.073
* Pt-1*,* Pt-2*,* …*,* Pt-10*,* Qt*	0.127	0.031	0.993	0.993	0.064	0.017	0.992	0.992
* Pt-1*,* Pt-2*,* …*,* Pt-10*,* Qt*,* Qt-1*,*…*,* Qt-5*	**0.061**	**0.012**	**0.998**	**0.998**	**0.032**	**0.006**	**0.998**	**0.998**
GPR-Kmeans (3-P*)								
* Pt-1*,* Pt-2*,* …*,* Pt-5*	0.698	0.319	0.790	0.792	0.344	0.221	0.770	0.781
* Pt-1*,* Pt-2*,* …*,* Pt-10*	0.646	0.313	0.820	0.822	0.330	0.219	0.787	0.799
* Pt-1*,* Pt-2*,* …*,* Pt-10*,* Qt*	0.108	0.032	0.995	0.995	0.077	0.023	0.988	0.998
* Pt-1*,* Pt-2*,* …*,* Pt-10*,* Qt*,* Qt-1*,*…*,* Qt-5*	**0.054**	**0.0091**	**0.999**	**0.999**	**0.026**	**0.004**	**0.999**	**0.999**
GPR-Kmeans (3-Q*)								
* Pt-1*,* Pt-2*,* …*,* Pt-5*	0.669	0.315	0.807	0.809	0.335	0.220	0.781	0.792
* Pt-1*,* Pt-2*,* …*,* Pt-10*	0.646	0.312	0.820	0.822	0.329	0.219	0.788	0.800
* Pt-1*,* Pt-2*,* …*,* Pt-10*,* Qt*	0.091	0.019	0.996	0.996	0.046	0.008	0.996	0.996
* Pt-1*,* Pt-2*,* …*,* Pt-10*,* Qt*,* Qt-1*,*…*,* Qt-5*	**0.058**	**0.0095**	**0.999**	**0.999**	**0.027**	**0.004**	**0.999**	**0.999**
GPR-Kmeans (2-P)								
* Pt-1*,* Pt-2*,* …*,* Pt-5*	0.886	0.417	0.662	0.672	0.456	0.301	0.594	0.630
* Pt-1*,* Pt-2*,* …*,* Pt-10*	0.831	0.408	0.702	0.713	0.451	0.298	0.604	0.643
* Pt-1*,* Pt-2*,* …*,* Pt-10*,* Qt*	0.092	0.020	0.996	0.996	0.047	0.009	0.996	0.996
* Pt-1*,* Pt-2*,* …*,* Pt-10*,* Qt*,* Qt-1*,*…*,* Qt-5*	**0.050**	**0.009**	**0.999**	**0.999**	**0.028**	**0.004**	**0.999**	**0.999**
GPR-Kmeans (2-Q)								
* Pt-1*,* Pt-2*,* …*,* Pt-5*	0.880	0.415	0.667	0.677	0.450	0.300	0.605	0.640
* Pt-1*,* Pt-2*,* …*,* Pt-10*	0.830	0.406	0.704	0.714	0.442	0.297	0.620	0.657
* Pt-1*,* Pt-2*,* …*,* Pt-10*,* Qt*	0.094	0.020	0.996	0.996	0.050	0.009	0.995	0.995
* Pt-1*,* Pt-2*,* …*,* Pt-10*,* Qt*,* Qt-1*,*…*,* Qt-5*	**0.068**	**0.010**	**0.998**	**0.998**	**0.029**	**0.004**	**0.998**	**0.998**

*3-P and 3-Q indicate 3 clusters with respect to precipitation and streamflow inputs, vice versa; bold numbers indicate the best model with the lowest RMSE, MAE and the highest NSE and R^2^ for each method.

Additionally, two input combinations of rainfall measured up to a tenth lag were used, again pointing to the difficulties in correctly predicting hourly streamflow. This implies that the models should include the prior runoff to improve forecasting accuracy. From Table 1, it should be noted that, five input values of rainfall from (*Pt-1* to *Pt-5*) were included for the first input combination; ten input values of rainfall from (*Pt-1* to *Pt-10*) were included for the second input combination, the third input combination is always the second combined with runoff measured at the time t (*Qt*). Among the P inputs, input combination (ii) provided better accuracy and therefore, after the 2nd combination, streamflow was added to this combination. Finally, the fourth input combination uses ten lag times of rainfall (*Pt-1* to *Pt-10*) and six lag times of runoff (*Qt*, to *Qt-1 to Qt-5*). This procedure was applied by following the previous studies^6,7^. Adnan et al.^6^ indicated that using similar lagged inputs has been effective for short-term runoff predictions, including for 1-, 6-, and 12-hour horizons. Our study introduces K-means clustering within the GPR model to structure the data into clusters that align with specific runoff patterns. This clustering enables the model to capture distinct hydrological conditions, which may be especially beneficial for extended lead times such as 24-hour forecasts, where single-lagged input methods can face limitations. Furthermore, we noted that, our first objective was to select an optimal number of input variables (i.e., features) for each algorithm and to conduct a comparative study between various proposed models. Therefore, we compared the four input combinations described above, and we ranked the models based on multiple calculated numerical performance criteria, i.e., the RMSE, MAE, R^2^ and NSE.

### Determination of best-input combination

Results for one-hour ahead forecasting are reported in Table 1. In general, the obtained results were within two distinguished limits, i.e., very poor to excellent forecasting accuracies, indicating, as expected, that selecting the best-input variables plays a critical role. The numerical performance values for each of the six models, i.e., with and without clusters, are presented and discussed hereafter. As expected, Table 1 shows that some of the poorest agreement between measured and forecasted hourly streamflow occurs in the first and second input combinations, where only rainfall data are selected as input variables. For the first and second input combinations, this disagreement is significant for the GPR and PCR models, and it is not apparent that the predicted hourly streamflow was poorly correlated with the measured one, having a high forecasting error and very low fitting capabilities, reflected by a very low R^2^ and NSE values are (poorest correlation among all datasets). During the testing stage, the two models, i.e., the GPR and PCR, appear to have failed completely to capture the nonlinear relationships influencing hourly streamflow having a high RMSE and MAE values within the range of 0.446 m^3^/s to 0.474 m^3^/s, and from 0.735 m^3^/s to 0.759 m^3^/s, respectively.

Furthermore, the low R^2^ and NSE values (below 0.150) indicated that the models generated substantial disagreement between measured and forecasted data, making the models challenging to compare. A tentative model performance improvement was done by combining the K-means clustering and the GPR model (GPR-K-means), using two and three clusters of rainfall and runoff. In general, an improvement in the forecasting accuracies becomes more apparent, especially using three clusters of rainfall (*3-P*) and runoff (*3-Q*). Aggregation of the data into two clusters (2-P) and (2-Q) will significantly reduce the forecasting errors; as such, the RMSE and MAE may be substantially more precise, reaching values of 0.297 m^3^/s and 0.442 m^3^/s, with improvement rates of approximately 39.864% and 33.408% compared to the models without clusters. Further improvement was gained using the three clusters and the GPR-K-means (*3-Q*) seem to be slightly more accurate compared to the GPR-K-means (*3-P*). More precisely, using the first input combination (i.e., *Pt-1 to Pt-5*), the GPR-K-means (*3-P*) and GPR-K-means (*3-Q*) are very similar both in forecasting errors (RMSE 0.220 m^3^/s and MAE 0.335 m^3^/s) and fitting capabilities (R^2^ 0.790 and NSE 0.780). The two models have identical performances for the second input combination (i.e., Pt-1 to Pt-10) but slightly improve with larger R^2^ and NSE values of approximately 0.800 and 0.788, respectively. Yet, the models based on the K-means clustering show substantially lower RMSE and MAE values and the biggest R^2^ and NSE values.

The assumptions within the data regarding the impact of the measured runoff at various lag times and the improvement in the forecasting accuracies, were investigated. To diagnose some of the probable improvements beyond the first and second input combinations, we included the runoff data as an input variable for the third and fourth input combinations. As seen in Table 1, for the third input combination, the *Qt* was combined with ten rainfall data (*Qt*,* Pt-1 to Pt-10*), while for the fourth input combination larger number of data were combined (*Qt*,* Qt-1 to Qt-5*,* Pt-1 to Pt-10*). According to Table 1, all models showed their numerical performances significantly improved. All models have lower RMSE and MAE, reaching the values of 0.004 m^3^/s and 0.027 m^3^/s, and both algorithms showed an obvious excellent fitting level between estimates and measured data, for which the R^2^ and NSE values were approximately equal to 0.999, exhibiting a firm agreement. The numerical comparison between the algorithms with and without the inclusion of runoff data revealed excellent improvement rates between in situ measured and estimated data: the RMSE and MAE values dropped from 0.735 m^3^/s to 0.029 m^3^/s and from 0.446 m^3^/s to 0.005 m^3^/s for a reduction over 96% and 98% obtained using the GPR model. From 0.751 m^3^/s to 0.032 m^3^/s and from 0.460 to 0.006 for a decrease over 95% and 98% obtained using the PCR model. Furthermore, the best improvement was obtained using the GPR-Kmeans (3-Q) for which the lowest RMSE (0. 027) and MAE (0.004) values were obtained. In conclusion to the first analysis conducted in the present study, the best input combinations were selected and compared in the next section for 1-hour ahead, 6-hour ahead, 12-hour ahead and 24-hours ahead. Details of the obtained results are depicted in Table 2.

**Table 2:** Training and validation statistics of ML methods in estimating streamflow different horizons.

Input combination	Validation				Testing			
	RMSE, m^3^/s	MAE, m^3^/s	NSE	*R* ^2^	RMSE, m^3^/s	MAE, m^3^/s	NSE	*R* ^2^
1-hour ahead								
GPR	0.055	0.010	0.998	0.998	0.029	0.005	0.998	0.998
PCR	0.061	0.012	0.998	0.998	0.032	0.006	0.998	0.998
GPR-Kmeans (3-P*)	0.054	0.0091	0.999	0.999	**0.026**	**0.004**	**0.999**	**0.999**
GPR-Kmeans (3-Q*)	0.055	0.010	0.998	0.998	0.029	0.005	0.998	0.998
GPR-Kmeans (2-P)	0.050	0.009	0.999	0.999	0.028	0.004	0.999	0.999
GPR-Kmeans (2-Q)	0.068	0.010	0.998	0.998	0.029	0.004	0.998	0.998
6-hour ahead								
GPR	0.624	0.120	0.832	0.842	0.289	0.049	0.837	0.854
PCR	0.549	0.118	0.870	0871	0.261	0.058	0.867	0.869
GPR-Kmeans (3-P*)	0.389	0.092	0.935	0.935	**0.176**	**0.039**	**0.940**	**0.942**
GPR-Kmeans (3-Q*)	0.436	0.093	0.918	0.918	0.191	0.035	0.929	0.930
GPR-Kmeans (2-P)	0.448	0.090	0.914	0.914	0.195	0.035	0.926	0.927
GPR-Kmeans (2-Q)	0.452	0.094	0.912	0.912	0.204	0.039	0.919	0.920
12-hour ahead								
GPR	0.898	0.218	0.651	0.653	0.434	0.098	0.632	0.649
PCR	0.919	0.235	0.635	0.638	0.444	0.125	0.616	0.625
GPR-Kmeans (3-P*)	0.552	0.138	0.869	0.869	**0.241**	**0.055**	**0.887**	**0.891**
GPR-Kmeans (3-Q*)	0.644	0.164	0.821	0.821	0.298	0.067	0.827	0.832
GPR-Kmeans (2-P)	0.717	0.160	0.779	0.779	0.316	0.065	0.806	0.810
GPR-Kmeans (2-Q)	0.763	0.185	0.749	0.749	0.356	0.080	0.752	0.763
24-hour ahead								
GPR	1.189	0.344	0.389	0.392	0.559	0.156	0.390	0.421
PCR	1.226	0.374	0.350	0.358	0.568	0.214	0.371	0.391
GPR-Kmeans (3-P*)	0.641	0.177	0.823	0.824	**0.271**	**0.076**	**0.857**	**0.859**
GPR-Kmeans (3-Q*)	0.739	0.219	0.765	0.765	0.320	0.093	0.801	0.810
GPR-Kmeans (2-P)	0.847	0.212	0.691	0.695	0.356	0.094	0.753	0.755
GPR-Kmeans (2-Q)	0.944	0.255	0.616	0.618	0.416	0.108	0.663	0.668

*3-P and 3-Q indicate 3 clusters with respect to precipitation and streamflow inputs, vice versa; bold numbers indicate the best model with the lowest RMSE, MAE and the highest NSE and R^2^ for each method; the results are for the input combination of *Pt-1*,* Pt-2*,* …*,* Pt-10*,* Qt*,* Qt-1*,*…*,* Qt-5*.

### Results of multi-step ahead hourly streamflow forecasting

The comparison between the models based on the best-input combination selected in the previous section is depicted in Table 2. According to Table 2, the six models, i.e., the GPR, the PCR, the GPR with two and three rainfall clusters (GPR-K-means (2-P) and (3-P)), and with two and three runoff clusters (GPR-K-means (2-Q) and (3-Q)), were compared for multi-step ahead hourly streamflow forecasting, i.e., 1-hour, 6-hour, 12-hour and 24-hour intervals. While all models performed similarly at the 1-hour forecast horizon with near-identical values for RMSE, MAE, R^2^, and NSE, the proposed GPR-K-means model showed a marked improvement over standalone GPR and PCR at the longer 24-hour horizon. In particular, the GPR-K-means (3-Q) model achieved the lowest RMSE (0.271 m^3^/s), the lowest MAE (0.076 m^3^/s), and the highest R² (0.859) and NSE (0.857), demonstrating an increased ability to capture nonlinear patterns and variability in streamflow dynamics at extended lead times. This improvement is significant because extended forecasts are valuable for effective watershed management and flood risk mitigation. In the 24-hour horizon, the GPR-K-means model’s superior performance highlights its potential as a more reliable model for longer-term predictions, providing essential insights and robust accuracy that the standalone GPR and PCR models lack over this timescale. Thus, the GPR-Kmeans model provides short-term accuracy and demonstrates resilience and precision in extended forecasts, a critical advantage in hydrological forecasting applications.

For the 6-hour ahead forecasting, Table 2 shows the statistical metrics for each model as well as the performances of the models using the K-means clustering algorithms. The results reported in Table 2 are significant for each model and clearly show that the forecasting accuracies still need to be more accurate compared to the 1-hour ahead forecasting horizon. There are notable differences in the calculated numerical performances between the two forecasting horizons. While, we can see that, the ensemble relationship between measured and forecasted streamflow remains strong, although with a somewhat lower R^2^ and NSE (i.e. compared to the 1-hour ahead). The RMSE and MAE values ranged from 0.176 m^3^/s to 0.289 m^3^/s and from 0.039 m^3^/s to 0.049 m^3^/s, respectively, while the R^2^ and NSE values ranged from 0.854 to 0.942 and from 0.837 to 0.940, respectively, and the best numerical performances were obtained using the GPR-K-means (3-P). By comparing the numerical performances derived from 1-hour ahead to those of 6-hour ahead we can see that the RMSE and MAE values were increased by 85.22% and 89.74%, respectively, while the R^2^ and NSE values were decreased by 5.7% and 5.9%, respectively. In general, the correlation between measured and modeled streamflow is quite good. However, the GPR-K-means (3-P) model tends to notably provide high forecasting errors compared to the results obtained for 1-hour ahead, and these differences can be appreciated more clearly by looking at the calculated RMSE and MAE values.

According to the results provided in Table 2, the various ML models continuously showed a dip in their performance for 12-hour ahead forecasting. More precisely, we can see that, the best performances were obtained by the GPR-K-means (3-P) with RMSE, MAE, R^2^ and NSE values of approximately 0.241 m^3^/s, 0.055 m^3^/s, 0.891 and 0.887, respectively. The GPR and PCR were the poorest models, having the biggest RMSE and MAE values, and the lowest R^2^ and NSE values. Comparison between the GPR-K-means (3-P) for 1-hour ahead and for 6-hour ahead revealed that, the RMSE and MAE values were increased by 89.21% and 92.72%, respectively, while the R^2^ and NSE values were decreased by 10.80% and 11.20%, respectively. Finally, for the 24-hour forecasting horizon, we can clearly see that the GPR and PCR completely failed to correctly estimate streamflow, showing very high RMSE and MAE, reaching the values of 0.568 m^3^/s, 0.214 m^3^/s, and very low R^2^ and NSE values (0.391 and 0.371, respectively). The best performances with the lowest RMSE and MAE were always obtained by the GPR-Kmeans (3-P) having many input variables, i.e., more than 16 input variables, including rainfall and runoff data. However, the results indicated that runoff data explained the most variability, but rainfall was the second most important set of features. This result is certainly related to the high autocorrelation between the successive streamflow lags. Conversely, the rainfall and runoff were poorly correlated, leading to poor forecasting accuracies. However, the GPR-Kmeans (3-P) model guaranteed the best performances for the 24-hour ahead horizon, exhibited lower RMSE (0.271 m^3^/s) and MAE (0.076 m^3^/s), and the higher R^2^ (0.859) and NSE (0.857) values. In conclusion, while the results showed some variation between the 12-hour and 24-hour horizons, the differences were more pronounced from the 1-hour to 12-hour horizons. As expected, the models achieved high accuracy with many predictor variables due to their structural complexity. However, given the short lead-time, it is likely that similar accuracy could be achieved with fewer inputs, suggesting a potential area for further optimization by reducing input variables without compromising performance. This result was the response to the poor correlation between rainfall and runoff.

**Fig. 4:**
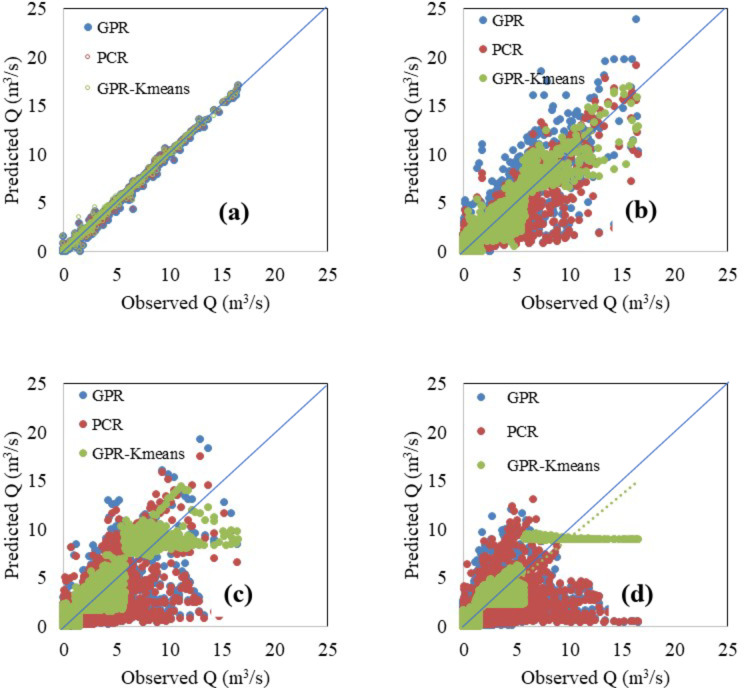
Comparison of GPR, PCR and GPR-K-means methods in predicting (**a**) 1 h ahead, (**b**) 6-hour ahead, (**c**) 12-hour ahead and (**d**) 24-hour ahead runoff – event 1 (blue line indicates the 1:1 line).

**Fig. 5:**
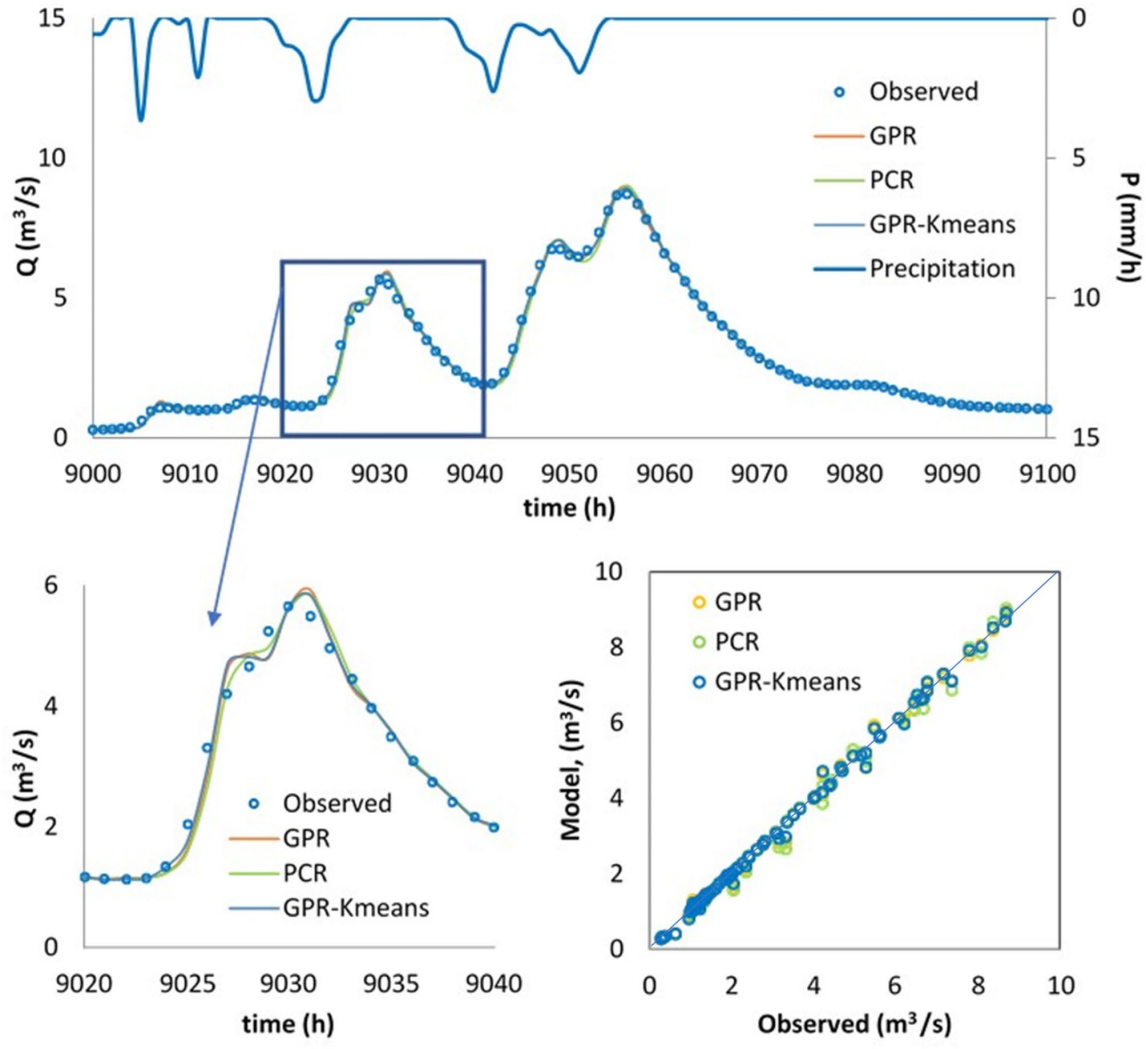
Comparison of GPR, PCR and GPR-Kmeans methods in predicting 1 h ahead runoff—event 1 (blue line in the scatterplot indicates the 1:1 line).

**Fig. 6:**
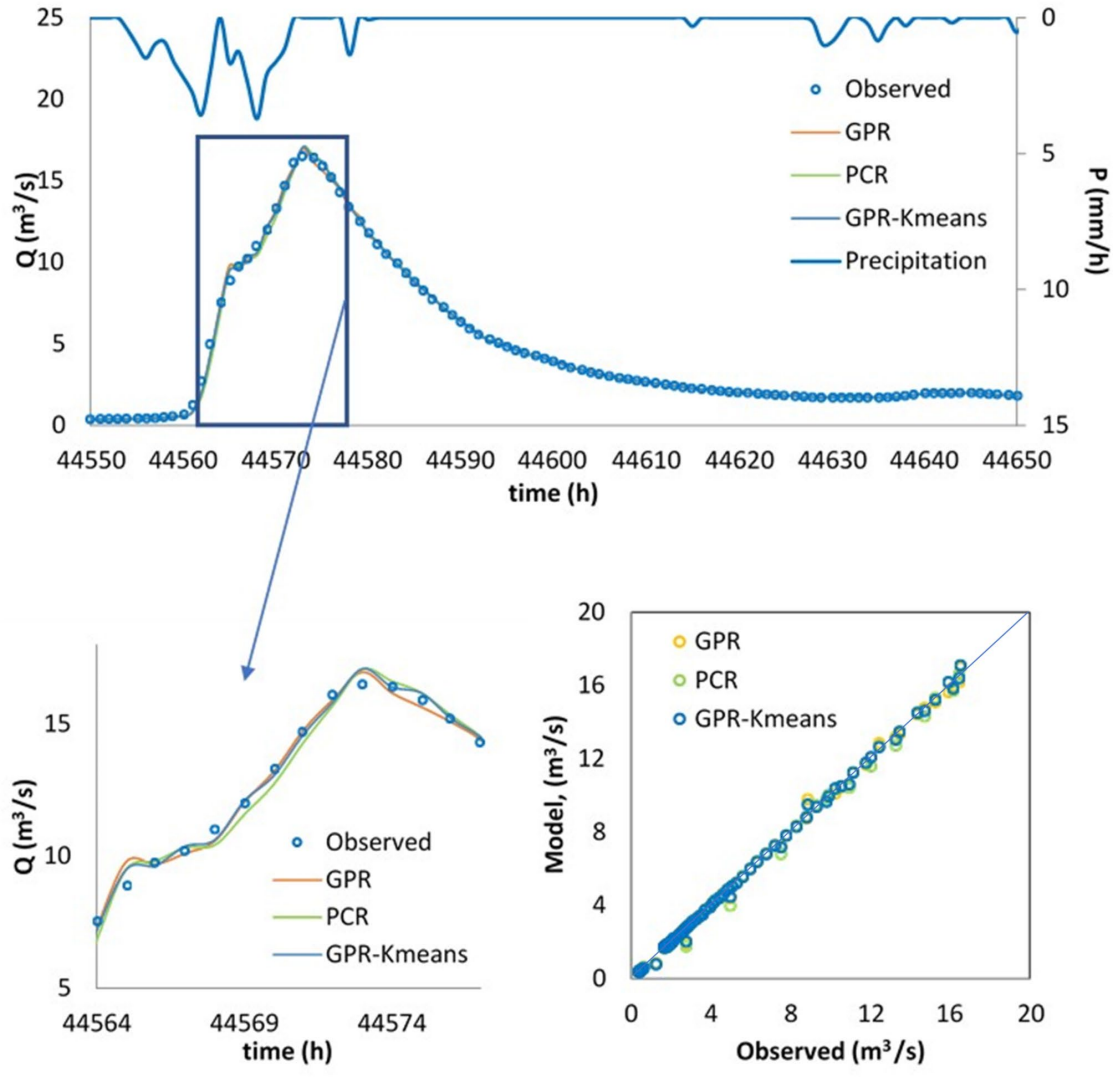
Comparison of GPR, PCR and GPR-Kmeans methods in predicting 1 h ahead runoff – event 2 (blue line in the scatterplot indicates the 1:1 line).

**Fig. 7:**
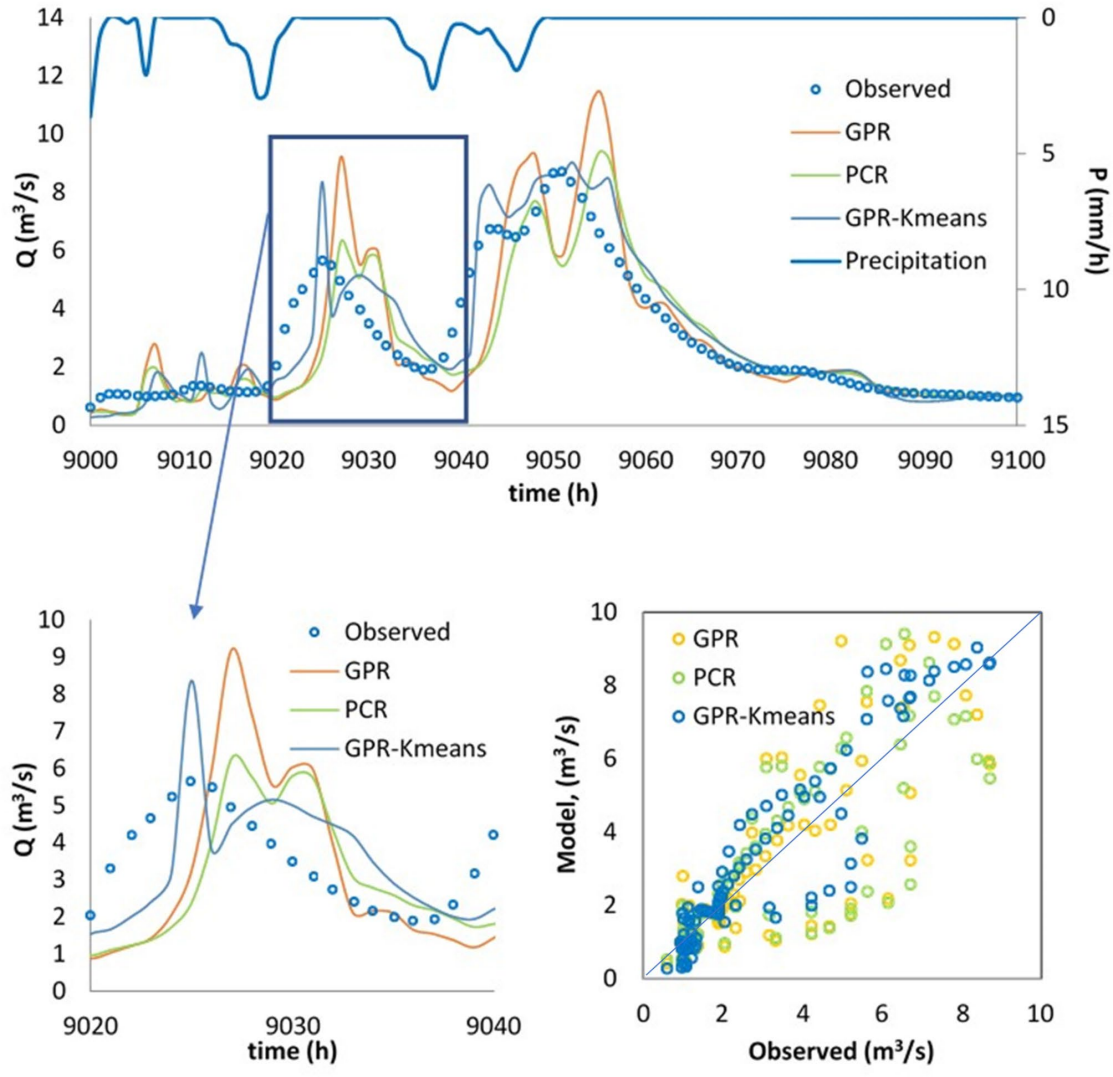
Comparison of GPR, PCR and GPR-Kmeans methods in predicting 6-hour ahead runoff – event 1 (blue line in the scatterplot indicates the 1:1 line).

**Fig. 8:**
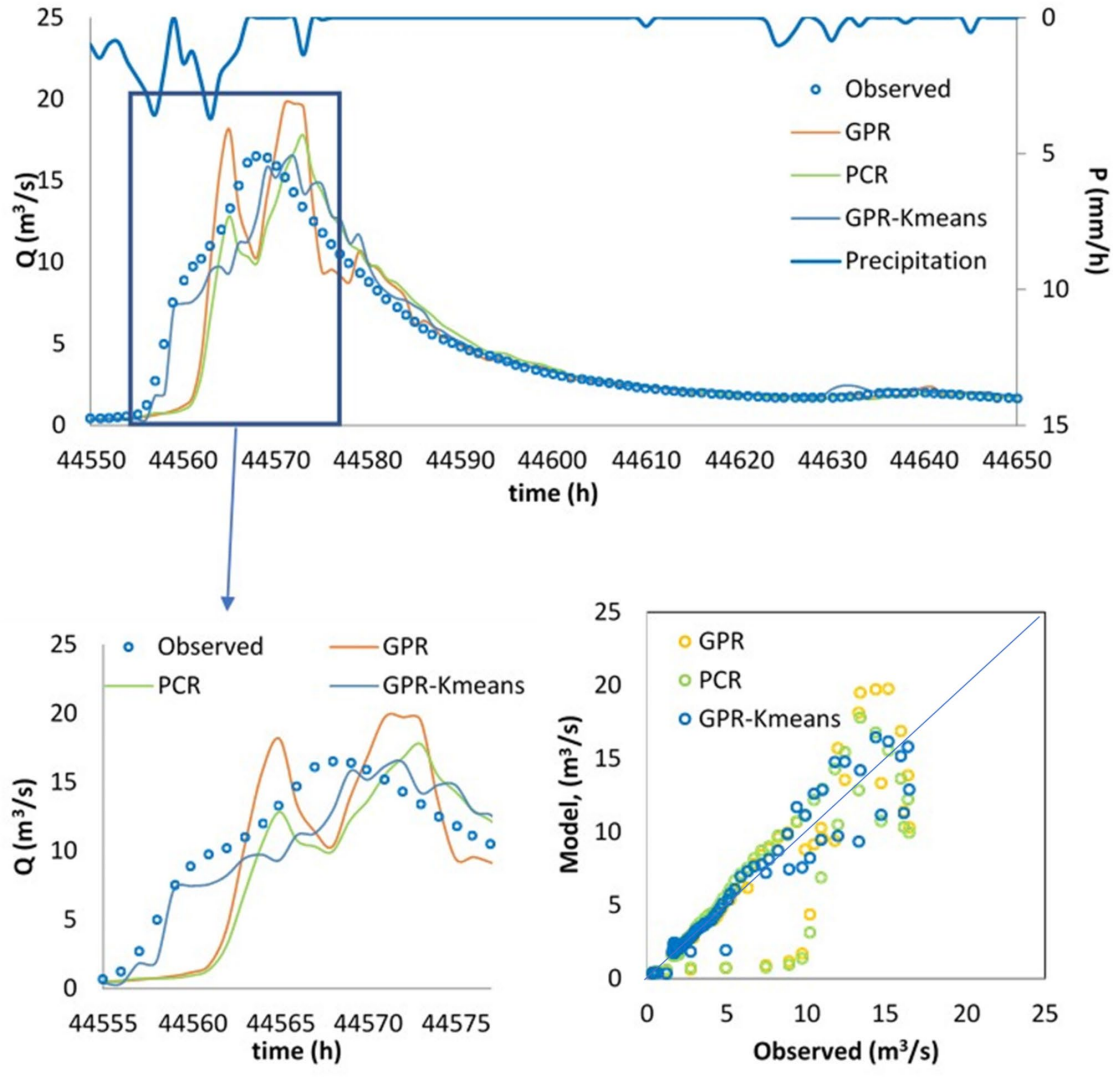
Comparison of GPR, PCR and GPR-Kmeans methods in predicting 6-hour ahead runoff – event 2 (blue line in the scatterplot indicates the 1:1 line).

**Fig. 9:**
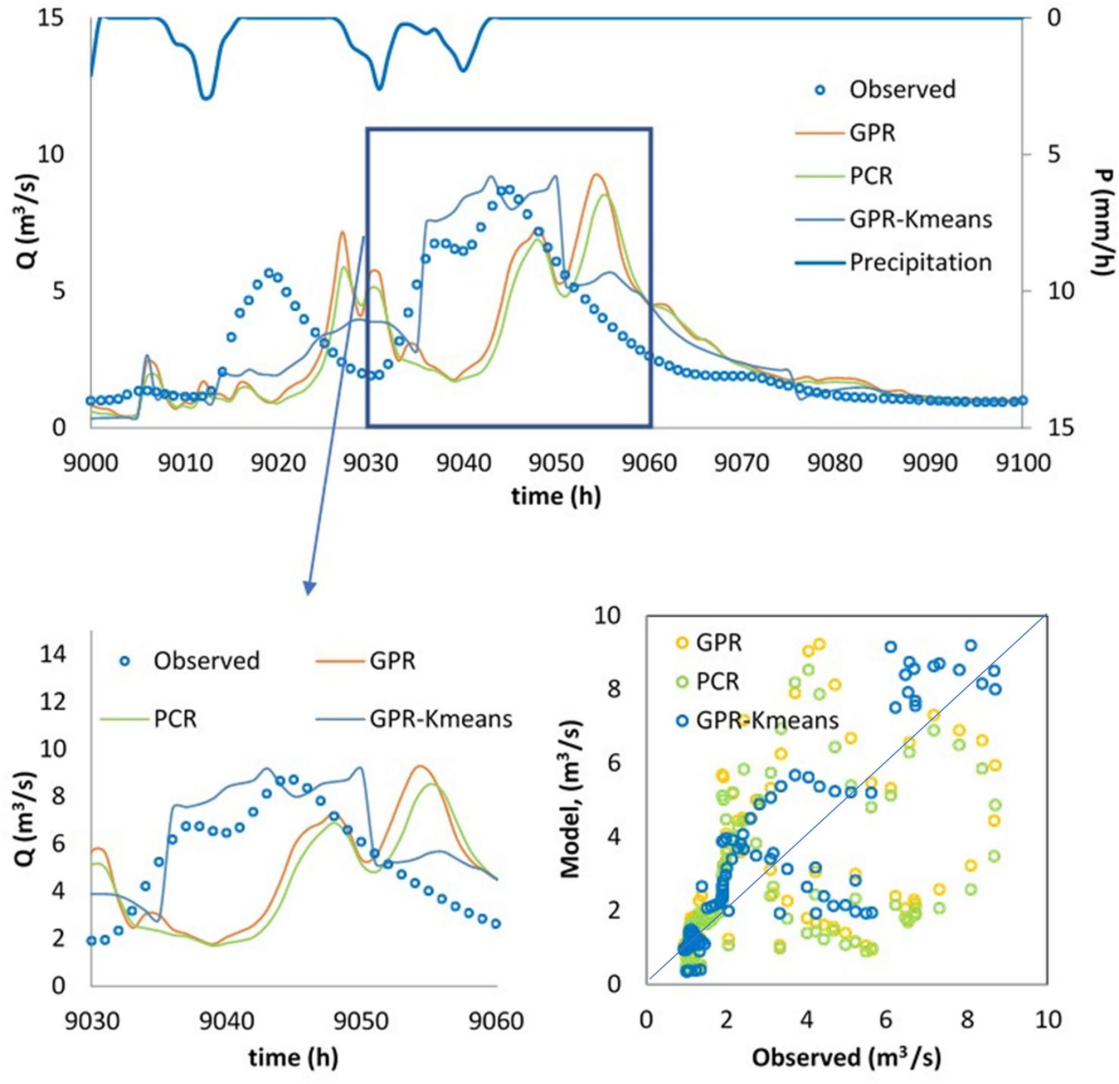
Comparison of GPR, PCR and GPR-Kmeans methods in predicting 12-hour ahead runoff – event 1 (blue line in the scatterplot indicates the 1:1 line).

**Fig. 10:**
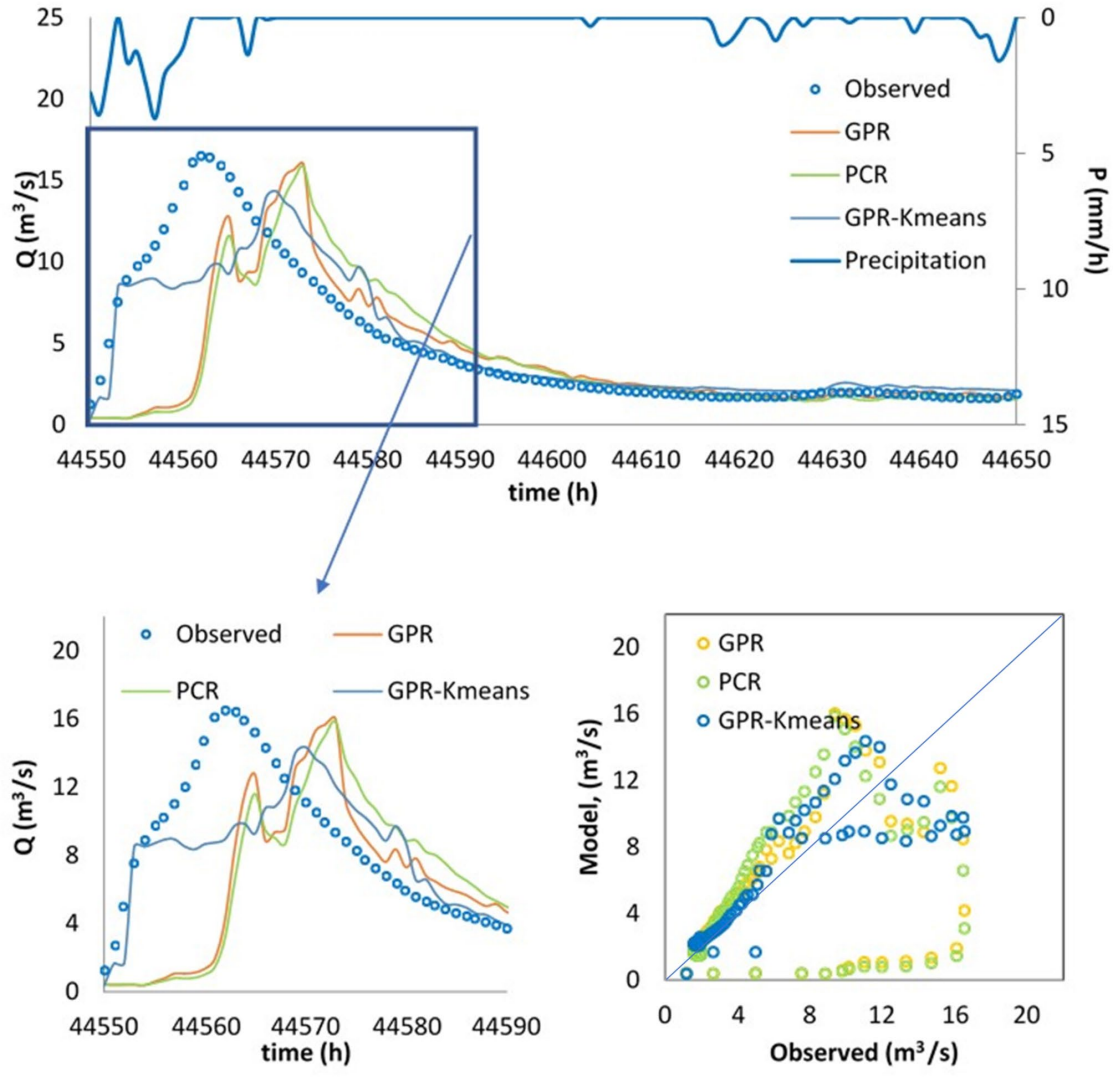
Comparison of GPR, PCR and GPR-Kmeans methods in predicting 12-hour ahead runoff – event 2 (blue line in the scatterplot indicates the 1:1 line).

**Fig. 11:**
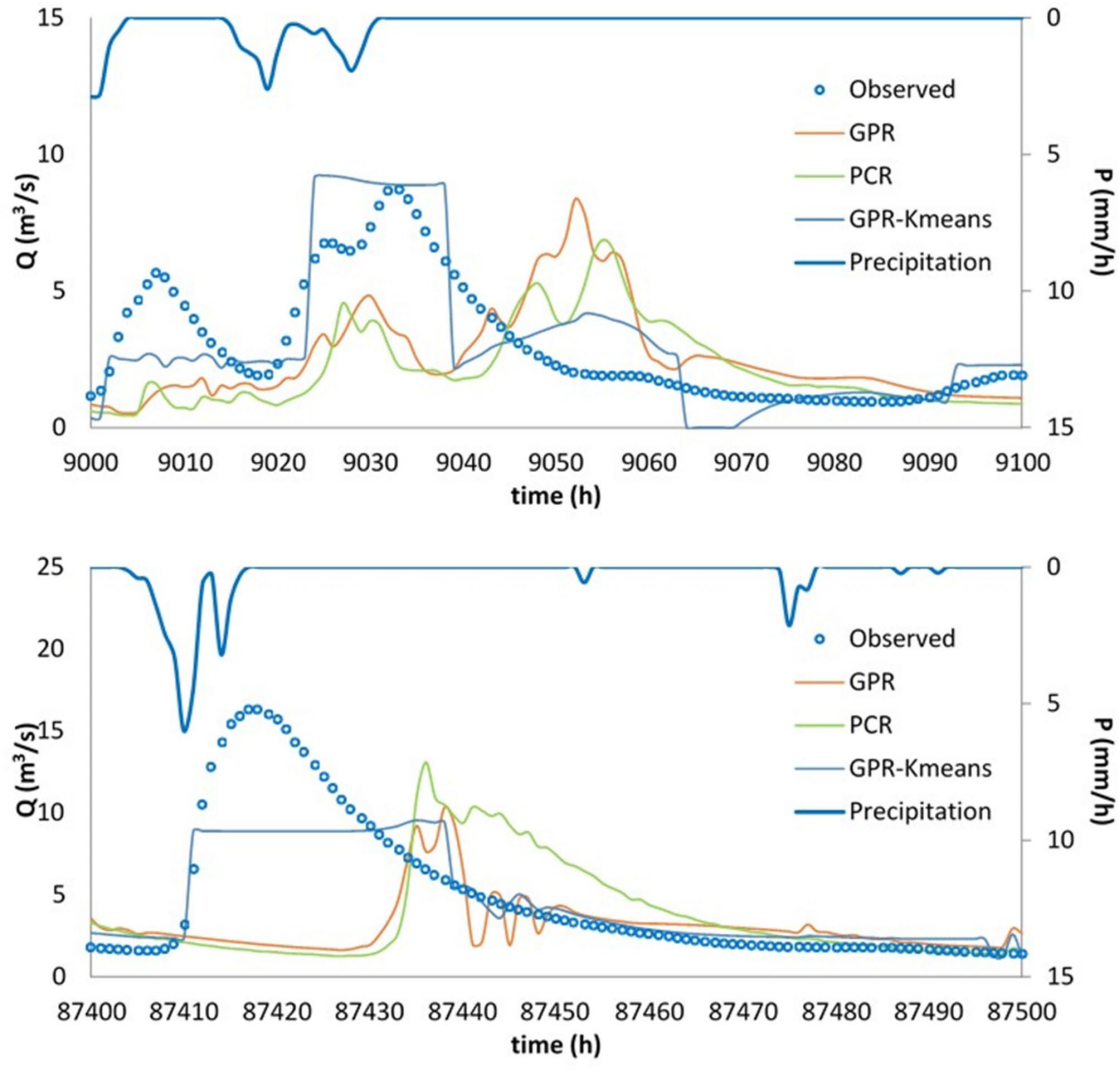
Comparison of GPR, PCR and GPR-Kmeans methods in predicting 24-hour ahead runoff – event 1 and event 2.

**Fig. 12:**
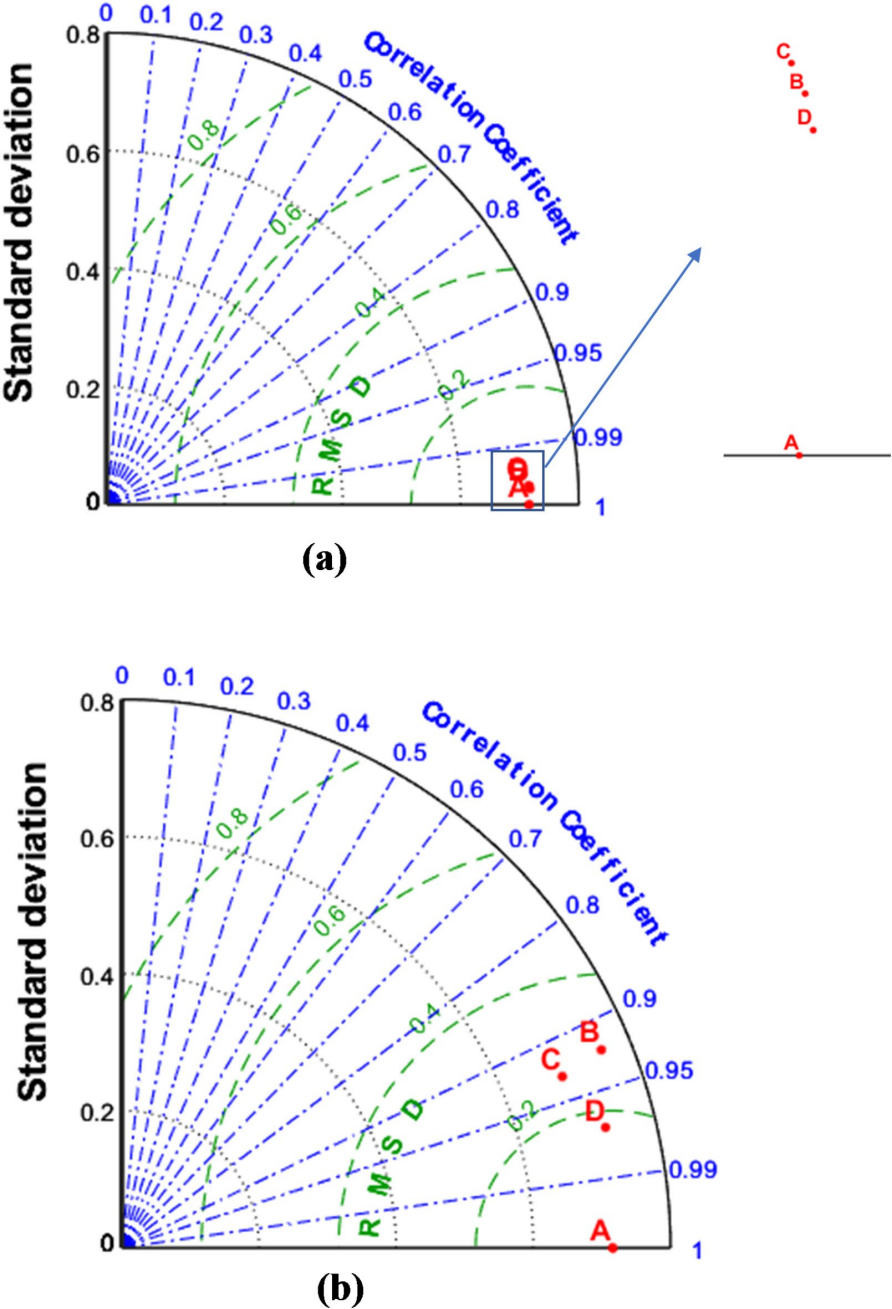
Taylor diagrams of the runoff estimation models for (**a**) 1-hour ahead and (**b**) 6-hour ahead. In the figure A: Observed runoff, B: GPR, C: PCR and D: GPR-Kmeans.

**Fig. 13:**
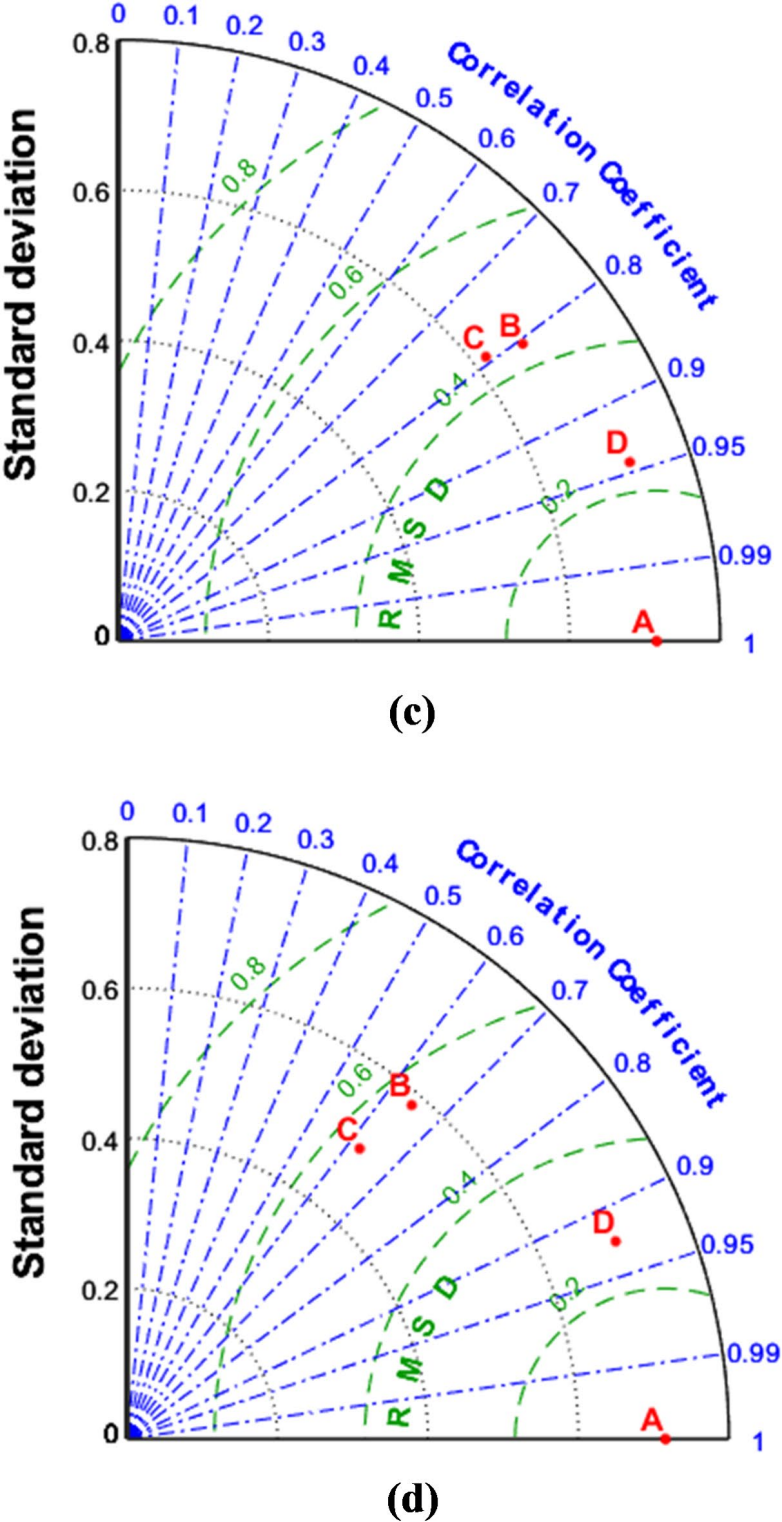
Taylor diagrams of the runoff estimation models for c) 12-hour ahead and d) 24-hour ahead. In the figure A: Observed runoff, B: GPR, C: PCR and D: GPR-Kmeans.

**Fig. 14:**
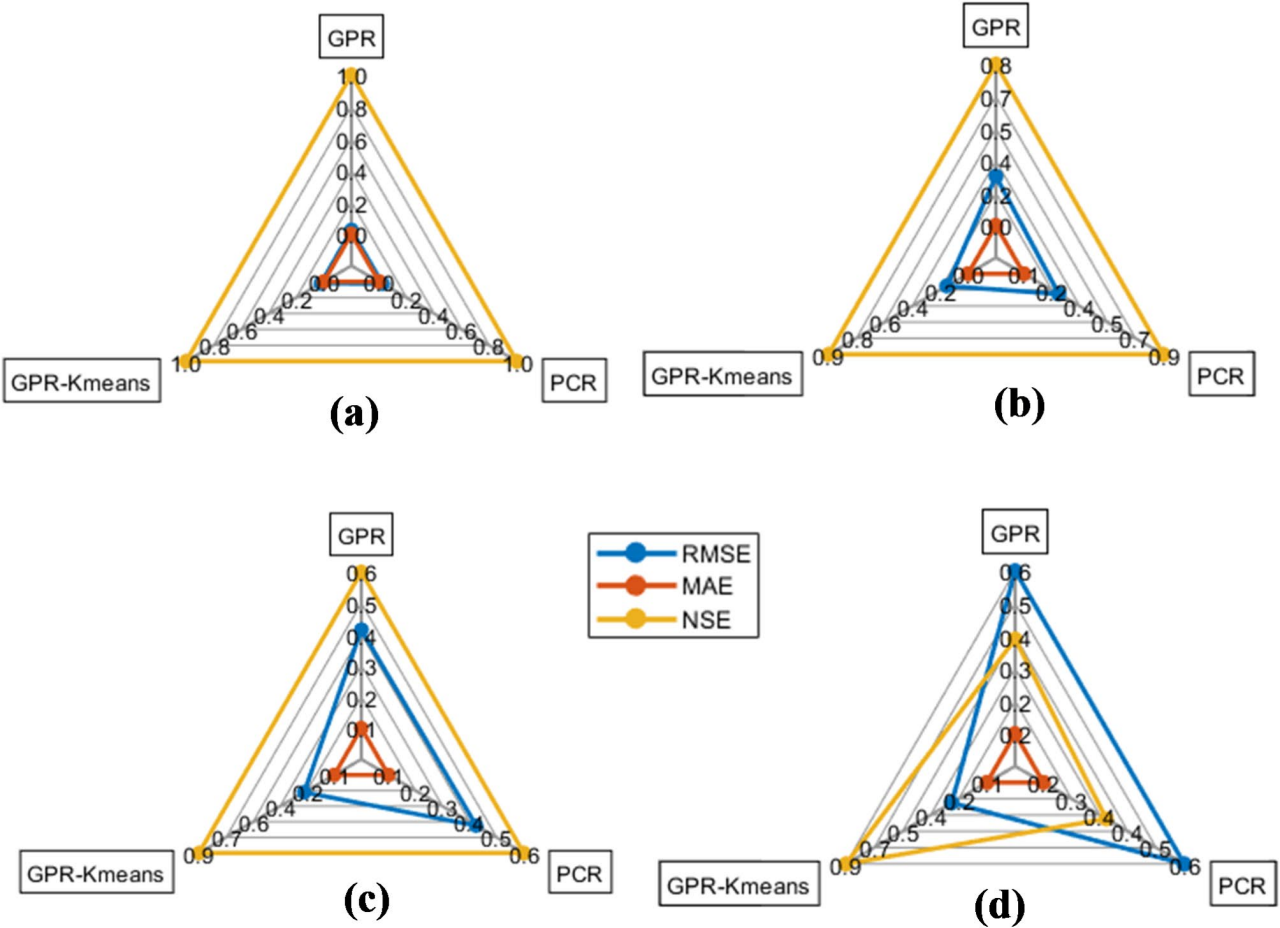
Spider plot of the runoff estimation models for (**a**) 1-hour ahead and (**b**) 6-hour ahead, (**c**) 12-hour ahead and (**d**) 24-hour ahead.

Finally, the best model, i.e., the GPR-Kmeans, was compared against the GPR and PCR for two distinguished events named Event 1 and Event 2. The three models were compared for 1-hour, 6-hour, 12-hour and 24-hour forecasting horizons, and the obtained results are reported in Table 3; Figs. 4, 5, 6, 7, 8, 9, 10, 11, 12, 13 and 14. According to Table 3; Fig. 4, there is a close correspondence between measured and modeled streamflow for a 1-hour forecasting horizon, and all models exhibited excellent forecasting performances for both Event 1 and Event 2. The GPR-K-means seem to be slightly more accurate having the biggest NSE (0.997) value and the poorest RMSE (0.120 m^3^/s) and MAE (0.098 m^3^/s) values for Event 1, and NSE (0.999) value and the poorest RMSE (0.153 m^3^/s) and MAE (0.069 m^3^/s) values for Event 2. As the forecasting horizon increases, the performances of the GPR and PCR models become very low, especially for Event 1. Within Event 1, the streamflow was poorly estimated for the 6-hour ahead forecasting horizon with an RMSE of 1.565m^3^/s (NSE 0.501) using the GPR, and an RMSE of 1.414 m^3^/s (NSE 0.592) using the PCR. At the same time, the performances of the GPR-Kmeans were relatively acceptable (RMSE 0.963 m^3^/s, MAE 0.715, NSE 0.811). However, for the 6-ahead forecasting horizon and within Event 2, it can be found from Table 3 that, the GPR and PC worked similarly with acceptable performances. This statement certainly reflects that the forecasting accuracy of streamflow is enhanced, despite the fact that the GPR-K-means always appears to be more accurate, having the biggest NSE (0.929), and the poorest RMSE (1.167 m^3^/s) and MAE (0.653 m^3^/s) values, respectively.

**Table 3:** Comparison of machine learning methods in modeling rainfall-runoff of various events.

Method	RMSE, m^3^/s	MAE, m^3^/s	NSE	RMSE, m^3^/s	MAE, m^3^/s	NSE
	Event 1			Event 2		
1-hour ahead						
GPR	0.132	0.070	0.997	0.168	0.071	0.999
PCR	0.158	0.091	0.995	0.220	0.100	0.998
GPR-Kmeans	0.120	0.098	0.997	0.153	0.069	0.999
6-hour ahead						
GPR	1.565	0.974	0.501	2.092	0.986	0.773
PCR	1.414	0.926	0.592	2.114	1.054	0.768
GPR-Kmeans	0.963	0.715	0.811	1.167	0.653	0.929
12-hour ahead						
GPR	2.252	1.586	− 0.033	3.879	2.066	0.198
PCR	2.292	1.594	− 0.070	4.151	2.324	0.081
GPR-Kmeans	1.281	0.925	0.666	2.101	1.259	0.765
24-hour ahead						
GPR	2.601	2.018	− 0.418	5.230	3.065	− 0.359
PCR	2.681	2.140	− 0.507	5.767	3.787	− 0.652
GPR-Kmeans	1.445	1.126	0.562	2.344	1.432	0.727

Furthermore, as the forecasting horizon increases, the model performances decrease and the poorer estimates were further pronounced for the 12-hours and 24-hour forecasting horizons. The results in Table 3 confirm that the proposed GPR-K-means model performs best at both the 12-hour and 24-hour horizons, with a notable advantage at 24 h. The evaluation metrics for both Event 1 and Event 2 indicate that integrating K-means clustering enhances the model’s ability to maintain robust accuracy over extended forecasts, which the standalone GPR and PCR models struggle to achieve. This performance consistency, especially at the 24-hour horizon, underscores the GPR-Kmeans model’s suitability for real-world applications requiring high accuracy and reliability across multiple timescales, making it an ideal choice for operational rainfall-runoff forecasting. The numerical performances of the GPR-Kmeans for Event 2 seem superior to those of Event 1, and the two remaining models, i.e. the GPR and the PCR, have failed to provide an acceptable level of forecasting accuracy.

Figures 4, 5, 6, 7, 8, 9, 10, 11, 12, 13 and 14 present the forecast curves of GPR-Kmeans, GPR and PCR models. Remarkably, the developed GPR-Kmeans models were superior and greater than the GPR and PCR models. Although GPR and PCR models can capture the nonlinearity of the measured streamflow for the 1-hour ahead forecasting, their forecast curves seem to have high deficiencies. However, the forecast curves generated by the GPR-Kmeans provided acceptable predictions at the major peak values. Figure 4 compares GPR, PCR, and the hybrid GPR-K-means model for predicting runoff at different lead times (1-hour, 6-hour, 12-hour, and 24-hour ahead) for event (1) The GPR-K-means model outperforms the other models across all lead times, particularly for 1- and 6-hour ahead forecasts. This suggests that clustering enhances the model’s robustness by segmenting data into hydrological patterns that make short-term predictions more reliable. All models exhibit a decline in predictive accuracy as the forecast horizon extends, with the highest scatter and deviations at the 24-hour lead time. This highlights the challenges of capturing complex runoff dynamics over longer periods, even with advanced machine learning techniques. Figures 5 and 6 provide a detailed comparison of the GPR, PCR, and the hybrid GPR-K-means models for 1-hour ahead runoff prediction during events 1 and (2) In both events, GPR-K-means consistently outperforms or performs as well as GPR and PCR. This indicates that the clustering approach in GPR-K-means enhances model adaptability to various hydrological conditions, allowing it to capture peak flows and recession limbs more accurately. For both events, GPR, PCR, and GPR-K-means perform well in the 1-hour ahead prediction context. The minimal differences suggest that these models are all suitable for short-term forecasting, where recent data has strong predictive power due to a close temporal correlation with runoff response. While both events show similar model performance, slight differences in model alignment around peaks and recessions highlight that GPR-K-means may provide a marginal advantage, especially for high-flow events (e.g., in Event 2, which has a larger peak). Figures 7 and 8 compare GPR, PCR, and GPR-K-means performance models for predicting 6-hour ahead runoff during events 1 and 2. In both events, the GPR-K-means model demonstrates enhanced predictive power, especially around peak flows and recession periods. The clustering step in GPR-K-means likely improves its ability to handle the complex and delayed hydrological responses characteristic of 6-hour lead times. All models, including GPR-K-means, show some loss of accuracy for higher flow values, as seen in the scatter plots. This is expected for longer lead times, where predicting extreme runoff events becomes more challenging. Figures 9 and 10 compare the GPR, PCR, and GPR-K-means model for 12-hour ahead runoff predictions during events 1 and 2. Predictive accuracy declines substantially at the 12-hour forecast horizon across all models, as the extended lead time adds complexity in capturing precise runoff patterns. GPR-K-means demonstrates a clear advantage over GPR and PCR in both events, especially in capturing general trends during peaks and recessions. This indicates that clustering improves the model’s ability to handle the complexity of delayed runoff responses. Despite its advantages, GPR-K-means still struggles to accurately predict higher flow values, as shown by the spread in the scatter plots. This suggests that while clustering enhances adaptability, it does not completely mitigate the difficulty of predicting extreme events at extended lead times. Figure 11 compares GPR, PCR, and GPR-K-means models for 24-hour ahead runoff predictions during events 1 (top panel) and 2 (bottom panel). Predictive accuracy decreases notably across all models at the 24-hour forecast horizon. This extended lead time makes it challenging for the models to accurately capture both peak and recession phases, as the system dynamics over a full day introduce more complexity. Despite the performance decline, GPR-K-means generally follows the overall trend better than GPR and PCR in both events, particularly during the recession phases. This suggests that the clustering step provides some robustness against the uncertainties of longer lead times. Figures 12 and 13 present Taylor diagrams for the runoff estimation models, comparing their performance for the 1-hour ahead (panel a), 6-hour ahead (panel b), 12-hour ahead (panel c) and 24-hour ahead (panel d) forecasts. These diagrams highlight the performance of GPR, PCR, and GPR-K-means models in terms of correlation, standard deviation, and centered root mean square difference (RMSD) relative to the observed runoff. Both diagrams illustrate that model accuracy declines as the forecast horizon extends from 1-hour to 6-hour, as evidenced by lower correlation coefficients and increased RMSD. This is a common trend in hydrological forecasting due to the inherent complexity and uncertainty in predicting runoff over longer periods. GPR-K-means consistently outperforms both GPR and PCR across the 1-hour and 6-hour forecasts. Its proximity to the observed runoff point (A) in both Taylor diagrams highlights its strength in capturing the observed variability and trend more accurately, likely due to the clustering component that improves adaptability to varying hydrological patterns. The 12-hour and 24-hour forecasts illustrate the anticipated decrease in model accuracy as the forecast horizon extends. Both figures show reduced correlations and increased RMSD across all models. Despite the performance drop at longer lead times, GPR-K-means consistently outperforms GPR and PCR. It maintains closer alignment with the observed runoff data, particularly in terms of variability and error, demonstrating the effectiveness of clustering in capturing complex hydrological patterns. Both GPR and PCR show considerable deviations from observed runoff in the 12-hour and 24-hour forecasts, reflecting limitations in accurately capturing variability and trend as lead times increase. These standalone models struggle with the complexity introduced by the longer prediction intervals. Figure 14 presents spider plots comparing the runoff estimation models (GPR, PCR, and GPR-K-means) across different forecast horizons: (a) 1-hour ahead, (b) 6-hour ahead, (c) 12-hour ahead, and (d) 24-hour ahead. Across all panels, the performance metrics (RMSE, MAE, NSE) indicate that model accuracy decreases as the forecast horizon extends. This decline is more significant for GPR and PCR, which show much larger errors and lower NSE values for the 12-hour and 24-hour forecasts. GPR-K-means consistently outperforms GPR and PCR across all forecast horizons. Its lower RMSE and MAE values, combined with higher NSE scores, suggest that the integration of clustering improves the model’s robustness and accuracy, especially in the short- to intermediate-term forecasts. Although GPR-K-means remains the most accurate model, its performance declines at the 24-hour horizon, indicating that additional improvements or supplementary input variables may be required for reliable long-term runoff predictions.

## Discussion

In the present study, by combining the K-means clustering algorithm and GPR, the hybrid GPR-K-means model guaranteed an enhanced ability to accurately forecast hourly streamflow several hours in advance. In order to quantitatively evaluate the improvements gained using the GPR-K-means compared to two standalone models, i.e., the GPR and PCR, an attempt was made to forecast hourly streamflow at 1-hour, 6-hour, 12-hour and 24-hour in advance. The comparative results reveal that the forecasting accuracy was significantly improved. However, an increase in the forecasting horizon from 1-hour to 24-hour, are accompanied by a decrease in the models’ performances. Apart from this, the proposed method is also applicable to other application scenarios. Apart from this, the proposed GPR-K-means model was also investigated for two events. As previous studies have shown, there is a great interest in understanding the feasibility of hourly streamflow forecasting, while there is not much published work on this subject. To the best of the authors’ knowledge, there is no existing research concerning the application of the hybrid GPR-K-means model for hourly streamflow forecasting.

The GPR-K-means model’s performance aligns well with similar studies that have applied Gaussian Process Regression and hybrid clustering techniques for streamflow forecasting, though it demonstrates unique advantages in forecast accuracy over extended horizons. For instance, Sun et al.^28^ implemented GPR for monthly streamflow forecasting across several U.S. hydrometric stations, finding that GPR outperformed traditional linear regression and artificial neural networks (ANNs) in predictive accuracy. While effective for monthly predictions, their standalone GPR approach did not explore clustering integration, which our study shows can significantly improve accuracy at shorter, hourly scales, particularly over extended forecast horizons.

Similarly, Ehteram et al.^25^ introduced a hybrid model combining convolutional neural networks (CNN), support vector machines (SVM), and GPR for monthly and daily rainfall prediction in Malaysia’s Terengganu River Basin. The CNN-SVM-GPR model achieved high accuracy for monthly predictions, yet their results indicated a performance decline in shorter forecasts, where our GPR-K-means model maintains accuracy, especially in hourly streamflow predictions. By employing K-means clustering with GPR, our model effectively addresses the nonlinearity and variability in short-term rainfall-runoff relationships, enhancing predictive capability without needing the additional deep learning layers used in Ehteram et al.‘s hybrid model^25^. Several studies have integrated clustering techniques into ML-based rainfall-runoff models to enhance predictive performance. For instance, Wu et al.^27^ applied a self-organizing map (SOM) to preprocess hydrological data before using a support vector regression model, achieving notable improvements in extreme streamflow predictions. However, unlike SOM, which relies on unsupervised learning and neural network structures, K-means clustering offers a computationally efficient alternative that partitions data into distinct, non-overlapping clusters, making it well-suited for Gaussian Process Regression (GPR). This study extends these earlier efforts by integrating K-means with GPR, a Bayesian regression technique that provides probabilistic predictions, thus improving both accuracy and uncertainty quantification in short-term rainfall-runoff forecasting.

Furthermore, Xiang et al.^55^ tested various ML methods, including GPR, Long Short-Term Memory (LSTM) networks, and LSTM-sequence-to-sequence models, for rainfall-runoff modeling at two watersheds in Iowa, USA. Their GPR model, however, exhibited a substantial decline in NSE (around − 2.88) for longer forecasts, particularly at 24 h, where our GPR-K-means model maintains strong performance. By incorporating K-means clustering, we address the predictive limitations Xiang et al.^55^ encountered, especially for extended forecasts, as our model clusters data into more structured groups, capturing complex rainfall-runoff patterns that single GPR models or sequence-based networks struggled to maintain. In summary, while standalone GPR and hybrid machine learning models with clustering have shown effectiveness in rainfall-runoff modeling, our GPR-K-means model uniquely balances short-term accuracy with resilience at extended forecast horizons. This study demonstrates that clustering adds interpretability and helps structure data variability, enhancing GPR’s predictive performance across multiple timescales. This approach is distinct in maintaining low RMSE and high NSE values over 24-hour forecasts, suggesting broader applications in hydrological forecasting where robust, multi-step predictions are critical.

The high forecasting accuracy obtained in the present study is notable, especially in comparison to several published works. However, this accuracy must also be balanced with an understanding of model uncertainty, particularly regarding the bias-variance tradeoff. While the GPR-Kmeans model’s complexity enables it to capture intricate patterns in the data, it may also increase variance, potentially affecting generalizability across different datasets or catchments. For example, Xiang et al.^55^ compared the LSTM, the LSTM sequence-to‐sequence (LSTM-seq2seq), the LSTM-seq2seq distributed, the SVM, the LASSO, and the GPR, for forecasting hourly streamflow at 24-hour ahead, at two Midwestern watersheds, namely, Clear Creek and Upper Wapsipinicon River in Iowa, USA. From the obtained results, it was found that the calculated NSE values between measured and forecasted hourly streamflow were approximately equal to 0.533(LASSO), 0.572 (SVM), -2.886(GPR), 0.453(LSTM), 0.649(LSTM-seq2seq), and 0.768 (LSTM-seq2seq distributed), respectively, indeed, all less than the 0.999 obtained using our GPR-Kmeans. Finally, in another study, Han and Morrison^56^ compared the LSTM, the MLPNN and the SVM, for forecasting hourly streamflow several hours in advance. For 1-hour ahead forecasting, NSE values of 0.680, 0.850, and 0.870 were obtained by the MLPNN, SVM, and LSTM, respectively, while for the 6-hour ahead forecasting, the same three models provided NSE values of 0.550, 0.840, and 0.870, respectively. These results indicated the high fitting capability of our GPR-Kmeans compared to the previous MLPNN, SVM, and LSTM models.

Compared to similar hybrid approaches, the GPR-K-means model offers a balanced complexity suited for short-term streamflow predictions without the need for dense, computationally intensive deep learning models. For example, Ehteram et al.^25^ combined CNN, SVM, and GPR for monthly and daily rainfall forecasting, achieving high accuracy for monthly predictions but showing decreased accuracy at finer timescales. The clustering step in GPR-K-means organizes data into meaningful hydrological groupings based on rainfall intensity and other conditions, enabling the model to retain performance over hourly scales without deep layers. This model’s structure makes it adaptable to variations within each cluster, avoiding the high computational costs and data needs associated with complex neural networks.

While the GPR-K-means model shows promising results, the current study is limited by its focus on a single, relatively small watershed. Future studies should extend this hybrid approach to larger, more diverse catchments to assess its scalability, as model performance can vary significantly with catchment size and data complexity^13,28^. Additionally, alternative clustering methods, such as Self-Organizing Maps (SOM), could be explored to determine their effectiveness in data pre-processing and clustering for rainfall-runoff modeling^27^. Such approaches may offer advantages over K-means in capturing more complex data patterns and reducing overfitting, thereby enhancing the robustness of the model across varied hydrological settings.

The GPR-K-means model’s complexity, while beneficial for capturing nonlinear rainfall-runoff relationships, introduces challenges related to the bias-variance tradeoff—a well-known issue in ML applications^6,25^. For example, while the model demonstrated strong predictive accuracy for 1-hour forecasting, the accuracy declined at longer horizons (12-hour and 24-hour), indicating potential variance increase due to model complexity. Similar observations are seen in studies using ensemble models like RF-GPR, where clustering helps mitigate overfitting and improve generalizability^29^. Future adjustments to the GPR-K-means model could balance this tradeoff to better suit varied catchment conditions, enabling more robust performance across different temporal scales.

While the GPR-K-means model outperformed other machine learning models in short-term predictions, traditional physically-based models such as SWAT^23^ and HEC-HMS^13^ offer strengths in interpretability and generalizability across diverse hydrological scenarios. Vilaseca et al.^23^ found that the RF model was more accurate than SWAT in calibration phases but observed that SWAT’s structured approach provided reliable forecasts in data-sparse conditions. Likewise, Sayed et al.^13^ demonstrated that although HEC-HMS may not match the predictive power of ML models like GEP and ANFIS, it remains effective for long-term, continuous predictions. These comparisons illustrate that GPR-K-means is especially beneficial for short-term forecasting in data-rich settings, while conceptual models retain advantages in broader applications.

A limitation of the GPR-K-means model is its reliance on high-resolution, long-term data, as clustering requires variability in rainfall-runoff patterns to ensure accurate predictions. This dependency poses challenges in data-sparse catchments, a common issue in ML applications^6,24^. Furthermore, the model’s success in the Orgeval watershed—a relatively small and uniform catchment—suggests its transferability to larger or more varied basins may be constrained. Similar challenges were observed by Mohammadi^1^, who highlighted that while ML models perform well in controlled environments, they may falter in regions with differing topographies or hydrological characteristics. As such, this model is particularly suitable for data-rich settings, with potential adjustments needed for broader applications.

This study contributes to the state-of-the-art by introducing a hybrid GPR-K-means model that addresses common ML limitations, particularly in interpretability and uncertainty quantification^25,28^. Unlike standalone ML approaches, this model integrates K-means clustering to create structured groups that align with rainfall-runoff patterns, aiding interpretability by associating predictions with specific hydrological conditions. Additionally, GPR’s Bayesian framework allows for uncertainty quantification, a benefit noted in studies using Bayesian or probabilistic approaches for hydrological forecasting^28^. This innovation shows promise for advancing short-term prediction models that maintain both accuracy and interpretability, a balance often overlooked in hydrological ML applications.

This study advances rainfall-runoff modeling by demonstrating that clustering can enhance both the interpretability and predictive accuracy of GPR-based models. Unlike other ML approaches, which often lack interpretability and require extensive data, GPR-K-means leverages structured groupings to make predictions that reflect specific hydrological conditions, thus aiding interpretability. This contribution addresses a research gap, particularly in machine learning applications for hydrology, where uncertainty quantification and model transparency remain challenges. Our findings indicate that GPR-K-means can serve as an effective tool for short-term forecasting in data-rich settings, but additional work is needed to refine its generalizability for broader hydrological applications.

The GPR-K-means model shows promise in the Orgeval watershed, yet its generalizability across larger, more varied catchments is unproven. Studies such as those by Sun et al.^28^ and Sayed et al.^13^ suggest that model performance can vary significantly with catchment characteristics, emphasizing the need for further evaluation across different hydrological settings. Future work could examine the model’s scalability by applying it to larger catchments and testing alternative clustering methods, like Self-Organizing Maps (SOM), as Wu et al.^27^ used successfully in streamflow predictions. Such extensions would offer insights into the robustness of the GPR-K-means model, potentially expanding its applicability beyond controlled environments to real-world hydrological systems.

## Conclusions

This study introduced a novel hybrid model that integrates Gaussian Process Regression (GPR) with K-means clustering (GPR-K-means) for short-term rainfall-runoff modeling. The model was evaluated against standalone GPR and Principal Component Regression (PCR) models across four forecasting horizons (1-hour, 6-hour, 12-hour, and 24-hour ahead) using hourly precipitation and streamflow data from the Orgeval watershed in France. This study offers key insights and contributions to the field of hydrological modeling, as summarized below:

- The GPR-K-means model significantly outperformed both standalone GPR and PCR models in forecasting accuracy, achieving Nash-Sutcliffe Efficiency (NSE) values of 0.999, 0.942, 0.891, and 0.859 for 1-hour, 6-hour, 12-hour, and 24-hour forecasts, respectively. These results demonstrate the model’s robustness in capturing the nonlinear relationship between precipitation and runoff and suggest its value in high-accuracy, short-term forecasting.
- Compared to other machine learning models reported in the literature, such as Long Short-Term Memory (LSTM), Support Vector Machines (SVM), and Random Forest (RF), the GPR-K-means model demonstrated superior predictive performance across all forecasting horizons. This superiority highlights the effectiveness of combining Bayesian regression with clustering techniques, which allows for both uncertainty quantification and enhanced interpretability, key limitations in traditional ML models for hydrology. This hybrid approach contributes a novel methodology to hydrological modeling, balancing prediction accuracy with practical insights into rainfall-runoff dynamics.
- While the GPR-K-means model proved effective for the Orgeval watershed, its reliance on high-resolution, long-term data may limit applicability in data-sparse catchments. Additionally, its current validation on a single, small watershed raises questions about scalability to larger or more complex catchments. Future research should evaluate the model’s transferability to diverse watersheds with varying hydrological characteristics and consider alternative clustering methods, such as Self-Organizing Maps (SOM), to enhance adaptability. Extending the model to handle multi-scale data (e.g., daily, weekly, monthly forecasts) and incorporating additional hydro-meteorological and topographical variables could improve its accuracy and applicability.
- The improved forecasting accuracy achieved by the GPR-K-means model has significant implications for flood mitigation, watershed management, and hydraulic infrastructure safety. By providing timely and accurate streamflow predictions, this model can support decision-makers in reducing risks to urban and rural infrastructure. With further validation, this approach could serve as a valuable tool in real-time monitoring systems, enhancing the resilience of water resource management strategies.

In conclusion, the proposed GPR-K-means model provides a practical, accurate, and interpretable approach for short-term rainfall-runoff forecasting. Its application to the Orgeval watershed demonstrates its potential as a robust tool in hydrological modeling, marking an advancement in integrating machine learning techniques within water resource management.

## Data Availability

The data presented in this study are available upon request from the corresponding author (contact ozgur.kisi@th-luebeck.de).
